# A Novel Family of [1,4]Thiazino[2,3,4-*ij*]quinolin-4-ium Derivatives: Regioselective Synthesis Based on Unsaturated Heteroatom and Heterocyclic Compounds and Antibacterial Activity

**DOI:** 10.3390/molecules26185579

**Published:** 2021-09-14

**Authors:** Vladimir A. Potapov, Roman S. Ishigeev, Lyudmila A. Belovezhets, Svetlana V. Amosova

**Affiliations:** A. E. Favorsky Irkutsk Institute of Chemistry, Siberian Division of the Russian Academy of Sciences, 1 Favorsky Str., 664033 Irkutsk, Russia; ishigeev@irioch.irk.ru (R.S.I.); belovezhets@irioch.irk.ru (L.A.B.); amosova@irioch.irk.ru (S.V.A.)

**Keywords:** annulation reactions, [1,4]thiazino[2,3,4-*ij*]quinolin-4-ium derivatives, 8-quinolinesulfenyl chloride, heterocycles, 4-pentenoic acid, 5-hexenoic acid, allyl halides

## Abstract

A novel family of [1,4]thiazino[2,3,4-*ij*]quinolin-4-ium derivatives was synthesized by annulation reactions of 8-quinolinesulfenyl chloride with unsaturated heteroatom and heterocyclic compounds. It was found that the reactions with 4-pentenoic and 5-hexenoic acids, allyl chloride and bromide, allyl cyanate and vinyl heterocyclic compounds (*N*-vinyl pyrrolidin-2-one and 1-vinylimidazole) proceeded in a regioselective mode but with the opposite regiochemistry. The reactions with vinyl heterocyclic compounds included electrophilic addition of the sulfur atom of 8-quinolinesulfenyl chloride to the β-carbon atom of the vinyl group. In the case of other substrates, the annulation proceeded with the attachment of the sulfur atom to the α-carbon atom of the vinyl group. The antibacterial activity of novel water-soluble compounds against *Enterococcus durans*, *Bacillus subtilis* and *Escherichia coli* was evaluated. Compounds with high antibacterial activity were found.

## 1. Introduction

**Figure 1:**
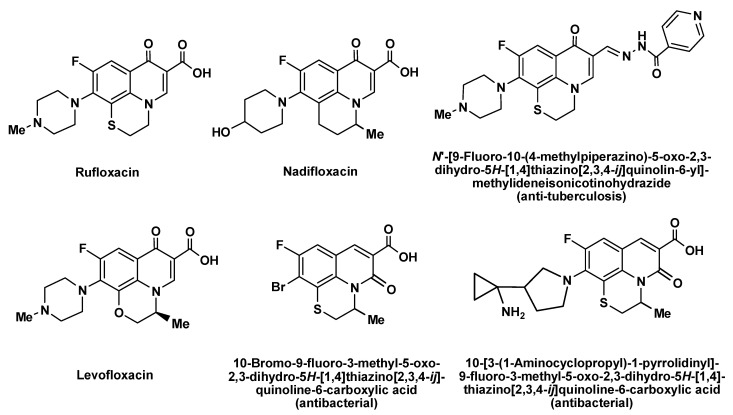
Known biologically active tricyclic quinoline compounds structurally related to the 2*H*,3*H*-[1,4]thiazino[2,3,4-*ij*]quinolin-4-ium scaffold (fluoroquinolone antibiotics [1,2,3,4,5], compounds with antibacterial [19] and anti-tuberculosis [20] activity).

Quinoline derivatives are used in the development of new drugs and exhibit a wide spectrum of biological activity [1,2,3,4,5]. Many medications contain the quinoline ring including antibacterial, antivirus and antimalarial (chloroquine, hydroxychloroquine, amodiaquine, primaquine) drugs [1,2,3,4,5]. The fluoroquinolone antibiotics (ciprofloxacin, levofloxacin, moxifloxacin, et al.) are one of most important classes of broad-spectrum bacteriocidals, which are very effective against both Gram-negative and Gram-positive bacteria [1]. A number of fluoroquinolone antibiotics (rufloxacin, levofloxacin, nadifloxacin) have a tricyclic core structure (Figure 1).

A combination of the quinoline scaffold with condensed sulfur-containing heterocycles has proven a fruitful approach in the development of new drugs [6,7]. Valuable examples of such combinations include penicillin and cephalosporin antibiotics, as well as the fluoroquinolone antibiotics prulifloxacin and rufloxacin (Figure 1). Levofloxacin and nadifloxacin represent antibiotics containing the quinoline scaffold condensed with six-membered cyclic structures (Figure 1).

A quinoline core structure fused with a thiazine heterocycle is a valuable scaffold for the development of derivatives with possible biological activity [8,9,10,11]. The 2*H*,3*H*-[1,4]thiazino[2,3,4-*ij*]quinolin-4-ium derivatives show various biological activities [12,13,14,15,16,17,18,19,20] including anticancer [18], antibacterial [19] and anti-tuberculosis [20] properties. The commonly used antibiotic rufloxacin can be also considered a 2*H*,3*H*-[1,4]thiazino[2,3,4-*ij*]quinolin-4-ium derivative (Figure 1).

**Scheme 1:**
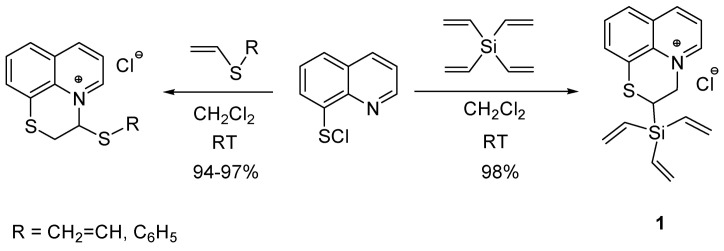
The annulation reactions of 8-quinolinesulfenyl chloride with vinyl sulfides and tetravinyl silane.

The development of a method for the efficient regioselective synthesis of novel heterocyclic and condensed organochalcogen compounds by cyclization and annulation reactions of chalcogen reagents is the focus of our research [21,22,23,24,25,26,27,28,29,30,31,32,33,34]. Recently we described the annulation reactions of 8-pyridinesulfenyl halides with functionalized alkenes and cycloalkenes affording a series of 2*H*,3*H*-[1,4]thiazino[2,3,4-*ij*]quinolin-4-ium derivatives in high yields [33,34]. For example, the annulation reactions with divinyl and vinyl phenyl sulfides proceeded with the attachment of the sulfur atom of 8-pyridinesulfenyl halides at the β-position of the vinylsulfanyl group, while the addition of the sulfur atom occurred at the α-carbon atom of the vinylsilyl moiety in the case of tetravinyl silane with the formation of 2-(trivinylsilyl)-2*H*,3*H*-[1,4]thiazino[2,3,4-*ij*]quinolin-4-ium chloride (**1**) (Scheme 1).

Despite some progress in the development of synthetic methods for the preparation of 2*H*,3*H*-[1,4]thiazino[2,3,4-*ij*]quinolin-4-ium derivatives [33,34,35,36,37,38,39,40,41], the annulation reactions of 8-quinolinesulfenyl halides with a number of vinylic heteroatom compounds (4-pentenoic acid, 5-hexenoic acid, allyl chloride and bromide, allyl cyanate, *N*-vinyl pyrrolidin-2-one, 1-vinylimidazole, ethyl and butyl vinyl ethers) have not been described in the literature. The synthesis of novel families of compounds with potential biological activity and evaluation of their antimicrobial properties represent urgent tasks. 

The goal of this research is the development of a method for the regioselective synthesis of a novel family of [1,4]thiazino[2,3,4-*ij*]quinolin-4-ium derivatives based on the annulation reactions of 8-quinolinesulfenyl chloride with unsaturated heteroatom and heterocyclic compounds (4-pentenoic acid, 5-hexenoic acid, allyl chloride and bromide, allyl cyanate, *N*-vinyl pyrrolidin-2-one, 1-vinylimidazole, 2,3-dihydrofuran, ethyl and butyl vinyl ethers) and the evaluation of their antibacterial activity.

## 2. Results and Discussion

**Scheme 2:**
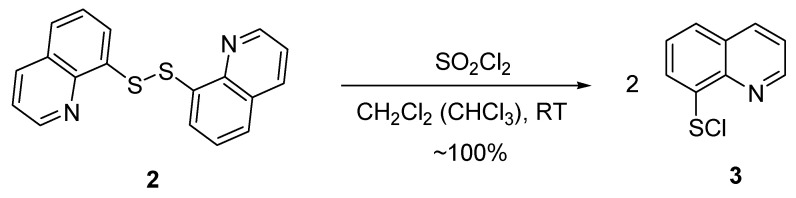
The generation of 8-quinolinesulfenyl chloride **3** from di(8-quinolinyl) disulfide **2** by the action of sulfuryl chloride.

The action of sulfuryl chloride on di(8-quinolinyl) disulfide (**2**) in methylene chloride or chloroform led to the generation of 8-quinolinesulfenyl chloride (**3**), which was used in situ without isolation in further reactions with unsaturated heteroatom compounds (Scheme 2). 

**Scheme 3:**
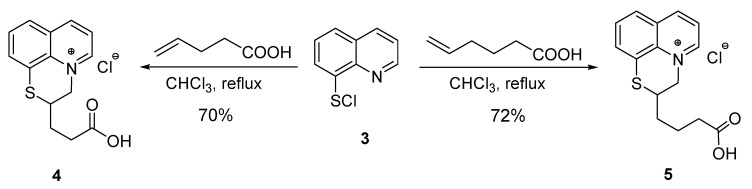
Synthesis of 2-(3-carboxyethyl)- and 2-(3-carboxypropyl)-2*H*,3*H*-[1,4]thiazino[2,3,4-*ij*]quinolin-4-ium chlorides **4** and **5** from sulfenyl chloride **3**, 4-pentenoic and 5-hexenoic acids.

Condensed water-soluble organic salts containing carboxyl function are very promising with respect to possible biological activity. We obtained [1,4]thiazino[2,3,4-*ij*]quinolin-4-ium derivatives based on the annulation reactions of 8-quinolinesulfenyl chloride **3** with terminal alkenes bearing the carboxylic acid function: 4-pentenoic and 5-hexenoic acids. When the annulation of 8-quinolinesulfenyl chloride with 4-pentenoic acid and 5-hexenoic acids was carried out in methylene chloride at room temperature, the reaction was found to be very sluggish. However, refluxing the reaction mixture in chloroform for 8 h made it possible to obtain 2-(3-carboxyethyl)- and 2-(3-carboxypropyl)-2*H*,3*H*-[1,4]thiazino[2,3,4-*ij*]quinolin-4-ium chlorides **4** and **5** with 70–72% yields (Scheme 3). Refluxing the reaction mixture in methylene chloride for 8 h gave products **4** and **5** with only 49–52% yields. 

Compounds **4** and **5** are light yellow water-soluble powders with a melting point above 160 °C.

**Scheme 4:**
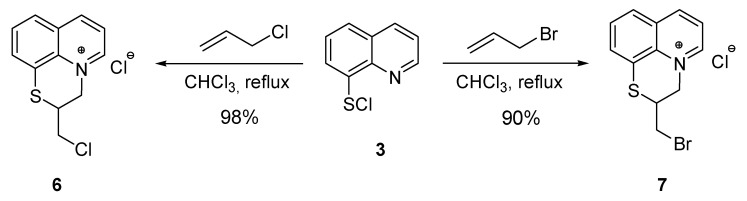
Synthesis of 2-(chloromethyl)- and 2-(bromomethyl)-2*H*,3*H*-[1,4]thiazino[2,3,4-*ij*]quinolin-4-ium chlorides **6** and **7** from allylchloride, allylbromide and sulfenyl chloride **3**.

Allylchloride and allylbromide were involved in the annulation reactions with 8-quinolinesulfenyl chloride **3**. By carrying out the reaction of sulfenyl chloride **3** with allylchloride and allylbromide under the same conditions as the synthesis of compounds **4** and **5** (refluxing the reaction mixture in chloroform for 8 h), 2-chloromethyl- and 2-(bromomethyl)-2*H*,3*H*-[1,4]thiazino[2,3,4-*ij*]quinolin-4-ium chlorides **6** and **7** were synthesized with 98% and 90% yields, respectively (Scheme 4).

The presence of carboxyl function and halogen atoms in structure of compounds **4**–**7** opens up opportunities for their functionalization by esterification, the nucleophilic substitution of halogen and other reactions. 

**Scheme 5:**
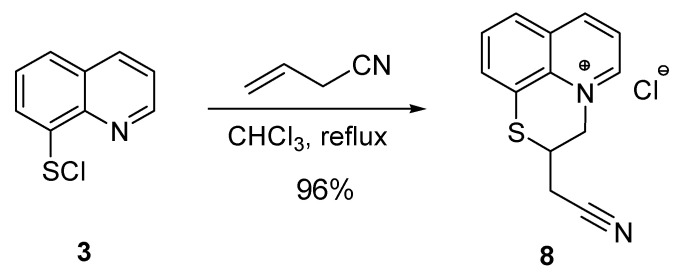
Synthesis of 2-cyanomethyl-2*H*,3*H*-[1,4]thiazino[2,3,4-*ij*]quinolin-4-ium chloride **8** from sulfenyl chloride **3** and allylcyanate.

The reaction of 8-quinolinesulfenyl chloride **3** with allyl cyanate was very sluggish at room temperature in methylene chloride. However, carrying out the reaction of sulfenyl chloride **3** with allyl cyanate for 8 h in refluxing chloroform made it possible to obtain 2-cyanomethyl-2*H*,3*H*-[1,4]thiazino[2,3,4-*ij*]quinolin-4-ium chlorides **8** with a 96% yield (Scheme 5). Refluxing the reaction mixture in methylene chloride for 8 h led to product **8** with only a 67% yield.

Compounds **6–8** are light yellow water-soluble powders with melting points of 138–140 °C, 162–164 °C and 183–185 °C, respectively.

The involvement of substrates bearing potentially pharmacophoric heterocycles in annulation reactions is important in terms of the possible manifestation of biological activity. 1-Vinylimidazole and *N*-vinyl pyrrolidin-2-one, which contain a vinyl group bonded to a nitrogen atom, were involved in the annulation reactions with 8-quinolinesulfenyl chloride **3**. The latter compound is an example of a heterocycle bearing a vinyl amide moiety in its structure. 

**Scheme 6:**
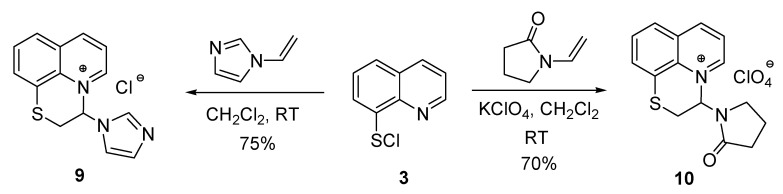
Synthesis of 3-(1*H*-imidazol-1-yl)-2*H*,3*H*-[1,4]thiazino[2,3,4-*ij*]quinolin-4-ium chloride **9** and 3-(2-oxopyrrolidin-1-yl)-2*H*,3*H*-[1,4]thiazino[2,3,4-*ij*]quinolin-4-ium perchlorate (**10**) from sulfenyl chloride **3** and 1-vinylimidazole and *N*-vinyl pyrrolidin-2-one.

The annulation reaction of 8-quinolinesulfenyl chloride **3** with 1-vinylimidazole was carried out at room temperature in methylene chloride, affording 3-(1*H*-imidazol-1-yl)-2*H*,3*H*-[1,4]thiazino[2,3,4-*ij*]quinolin-4-ium chloride (**9**) with a 75% yield (Scheme 6). 

Under the same conditions, the annulation reaction of 8-quinolinesulfenyl chloride **3** with *N*-vinyl pyrrolidin-2-one gave the annulation products with a 59% yield along with some by-products. It was found that this reaction proceeded more efficiently and selectively in the presence of potassium perchlorate.

3-(2-Oxopyrrolidin-1-yl)-2*H*,3*H*-[1,4]thiazino[2,3,4-*ij*]quinolin-4-ium perchlorate (**10**) was obtained with a 70% yield by the reaction of 8-quinolinesulfenyl chloride **3** with *N*-vinyl pyrrolidin-2-one in the presence of an equimolar amount of potassium perchlorate (Scheme 6).

Attempts were made to increase the yields by refluxing the reaction mixture in methylene chloride or chloroform. This made it possible to obtain products **9** and **10** with 90–94% yields; however, the selectivity of the reactions decreased, and compounds **9** and **10** were contaminated with by-products (6–10%), from which it was difficult to separate the target compounds. 

**Scheme 7:**
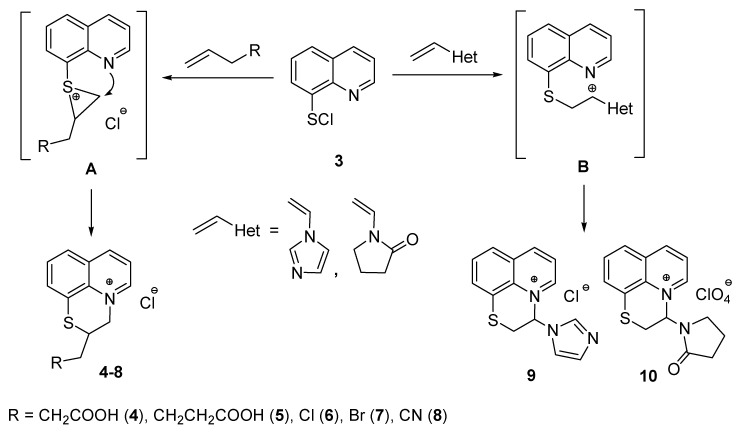
Directions of the reactions of sulfenyl chloride **3** with unsaturated heteroatom and heterocyclic compounds.

The reactions with 4-pentenoic and 5-hexenoic acids, allylchloride, allylbromide and allyl cyanate included the electrophilic addition of the sulfur atom from sulfenyl chloride **3** to the α-carbon atom of the vinyl group (“anti-Markovnikov direction”), while the annulation reactions with *N*-vinyl pyrrolidin-2-one and 1-vinylimidazole proceeded with the attachment of the sulfur atom to the β-carbon atom of the vinyl group (“Markovnikov direction”). We presume that the reactions of sulfenyl chloride **3** with *N*-vinyl pyrrolidin-2-one and 1-vinylimidazole proceed via linear intermediates **B** (Scheme 7) which are stabilized by the nitrogen atom (the nitrogen atom’s ability to stabilize adjacent carbocation is well known [42]). 

It is known that the electrophilic addition of sulfenyl chlorides [43,44,45,46,47,48,49,50,51,52] to linear 1-alkene leads predominantly to anti-Markovnikov products [43,44,45,46] and thiiranium cations are regarded as intermediates in these reactions [43,44,45,46,47,48]. In the cases of 4-pentenoic and 5-hexenoic acids, allylchloride, allylbromide and allyl cyanate, there are no heteroatoms (adjacent to the double bond) which could stabilize the intermediates, and the reactions take place via thiiranium intermediate **A**. Taking into account the steric factor, the nucleophilic attack of the nitrogen atom of the quinoline ring occurs at the unsubstituted carbon atom of thiiranium intermediate **A** and this course determines the “anti-Markovnikov direction” of the reactions (Scheme 7).

**Scheme 8:**
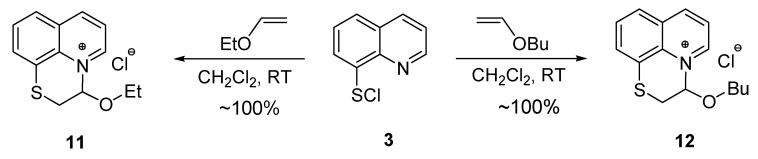
Synthesis of 3-ethoxy- and 3-butoxy-2*H*,3*H*-[1,4]thiazino[2,3,4-*ij*]quinolin-4-ium chlorides **11** and **12** from sulfenyl chloride **3** and ethyl vinyl and butyl vinyl ethers.

Vinyl ethers are promising substrates for annulation reactions due to the high reactivity of these compounds in electrophilic additions. The reactions of sulfenyl chloride **3** with ethyl vinyl and butyl vinyl ethers proceeded smoothly at room temperature in methylene chloride, producing 3-ethoxy- and 3-butoxy-2*H*,3*H*-[1,4]thiazino[2,3,4-*ij*]quinolin-4-ium chlorides **11** and **12** in quantitative yields (Scheme 8).

Like the synthesis of products **9** and **10**, the reactions of sulfenyl chloride **3** with ethyl vinyl and butyl vinyl ethers are believed to occur via linear intermediates (similar to intermediate **B**, Scheme 7), which are stabilized by the oxygen atom (the oxygen atom exhibits a strong ability to stabilize adjacent carbocation [53]). 

**Scheme 9:**
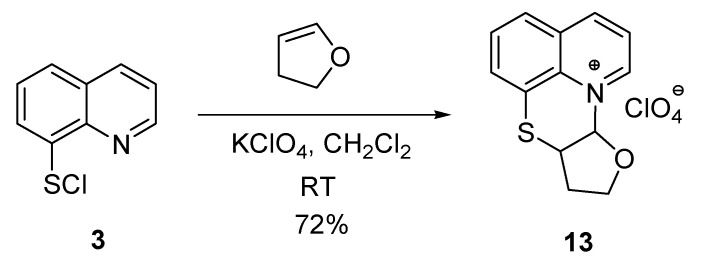
Synthesis of 7a*H*,8*H*,9*H*,10a*H*-furo[2′,3′:5,6][1,4]thiazino[2,3,4-*ij*]quinolin-11-ium perchlorate (**13**) from sulfenyl chloride **3**, 2,3-dihydrofuran and potassium perchlorate.

Finally, based on the reaction of sulfenyl chloride **3** with cyclic vinyl ether, 2,3-dihydrofuran, we synthesized the condensed four-membered heterocycle **13**, which is of interest for evaluation of antibacterial activity and comparison with the antibacterial properties of products **11** and **12**, obtained from ethyl vinyl and butyl vinyl ethers. The reactions of sulfenyl chloride **3** with 2,3-dihydrofuran was carried out in the presence of an equimolar amount of KClO_4_ at room temperature in methylene chloride, leading to perchlorate **13** with a 72% yield (Scheme 9). 

Similarly to the reactions with ethyl vinyl and butyl vinyl ethers (Scheme 8), synthesis of compound **13** was regioselective and the sulfur atom of sulfenyl chloride **3** bonded to the β-carbon atom of the vinyloxy group.

The antibacterial activity of the synthesized compounds was evaluated. The minimal inhibitory concentration (MIC) was determined using the broth standard microdilution method [54]. 

Compounds **1**, **4**–**13** were tested in vitro for antibacterial activity against bacterial strains of gram-positive *Enterococcus durans* B-603, *Bacillus subtilis* B-406 and gram-negative *Escherichia coli* B-1238 (the bacterial strains were taken from the All-Russian Collection of Microorganisms) and the obtained results were compared to the activity of standard aminoglycoside antibiotic gentamicin (the minimal inhibitory concentrations are 25, 50 and 100 μg/mL against *E. durans*, *B. subtilis* and *E. coli*, respectively). The obtained results are presented in the Table 1.

The activities of compounds **4** and **5**, which differ only in one CH_2_ group, are significantly different. Compound **5,** with its longer carbon chain, exhibited considerably higher activity against gram-positive *E. durans* and *B. subtilis* and is superior to antibiotic gentamicin in this respect (Table 1). 

Compounds **6** and **7** differ only in the halogen atom. Bromo-containing compound **7** was 40 times more effective than its chlorine analogue **6** against *E. durans*. However, product **6** was the most effective among the obtained compounds against gram-negative bacteria *E. coli*. Silicon-containing product **1** and compound **8** showed low activity. Compound **9** exhibited average activity against all tested bacteria (Table 1).

The comparison of compounds **11**–**13** revealed higher activity in products **11** and **13** (obtained from ethyl vinyl ether and 2,3-dihydrofuran), at levels which were superior to the activity of gentamicin against gram-positive bacteria. 

The highest activity was shown by product **10** (obtained from *N*-vinyl pyrrolidin-2-one), which significantly exceeded the activity of gentamicin and all obtained compounds against gram-positive bacteria and was more than a hundred times superior to this antibiotic against *B. subtilis* (Table 1).

The structural assignments of synthesized compounds were made using ^1^H and ^13^C-NMR spectroscopy, including two-dimensional experiments (Supplementary Materials containing examples of NMR spectra are available online), and confirmed by elemental analysis. 

The products with the opposite regiochemistry show the characteristic signals of carbon atoms bonded with a charged nitrogen (N^+^) atom and a sulfur atom. The number of protons (one or two) bonded to the carbon atoms adjacent to the charged nitrogen atom and to the sulfur atom is important (the number of protons is determined by NMR experiments). For example, the CHS moiety and the CH_2_N^+^ methylene group manifested themselves in the regions of 32–43 ppm and 58–64 ppm, respectively, in the ^13^C-NMR spectra of compounds **4**–**8** (the products derived from anti-Markovnikov addition of the sulfur electrophile to the double bond). Signals of the one-proton-containing OCHN^+^ moiety were observed in the downfield region of 91–92 ppm in the ^13^C-NMR spectra of compounds **11**–**13** (the products derived from Markovnikov addition of the sulfur electrophile to the double bond). 

## 3. Experimental Section

### 3.1. General Information

The ^1^H (400.1 MHz) and ^13^C (100.6 MHz) NMR spectra were recorded on a Bruker DPX-400 spectrometer (Bruker BioSpin GmbH, Rheinstetten, Germany) in 2–5% solution in D_2_O, DMSO-*d*_6_, methanol-*d*_4_ or acetone-*d*_6._ ^1^H and ^13^C chemical shifts (δ) were reported in parts per million (ppm), relative to tetramethylsilane (external) or to the residual solvent peaks of D_2_O (δ = 4.79), acetone-*d*_6_ (δ = 2.05 and 29.84 ppm), methanol-*d*_4_ (δ = 3.31 and 49.0 ppm) and DMSO-*d*_6_ (δ = 2.50 and 39.52 ppm for ^1^H and ^13^C NMR, respectively). The term “quino” in spectral data indicates belonging to the quinoline ring. The elemental analysis was performed on a Thermo Scientific FLASH 2000 Organic Elemental Analyzer (Thermo Fisher Scientific Inc., Milan, Italy). Melting points were determined on a Kofler Hot-Stage Microscope PolyTherm A apparatus (Wagner & Munz GmbH, München, Germany). Absolute solvents were used in the reactions.

### 3.2. Synthesis of Compounds ***4**–**8***

*2-(3-Carboxyethyl)-2H,3H*-[1,4]*thiazino[2,3,4-ij]quinolin-4-ium chloride* (**4**). A solution of sulfuryl chloride (0.076 g, 0.56 mmol) in chloroform (10 mL) was added dropwise to a solution of di(8-quinolinyl) disulfide (0.180 g, 0.56 mmol) in chloroform (10 mL), and the mixture was stirred for 10 min at room temperature. A solution of pentenoic acid (0.112 g, 1.12 mmol) in chloroform (10 mL) was added dropwise, and the reaction mixture stirred for 1 h at room temperature and 8 h at reflux temperature. After cooling in the refrigerator, the formed precipitate was filtered off and dried in a vacuum, producing the product (0.232 g, 70% yield) as a yellow powder, mp 170–172 °C. 

^1^H-NMR (400 MHz, D_2_O): δ 1.80–1.90 (m, 1H, CH_2_), 2.13–2.22 (m, 1H, CH_2_), 2.65 (t, *J* = 7.2 Hz, 2H, CH_2_), 3.85–3.86 (m, 1H, SCH), 5.07 (dd, *J* = 14.2, 6.8 Hz, 1H, NCH_2_), 5.31 (d, *J* = 14.3 Hz, 1H, NCH_2_), 7.74–7.77 (m, 1H, C_q__uino_), 7.93–7.94 (m, 1H, C_q__uino_), 8.00–8.04 (m, 2H, C_q__uino_), 9.06–9.12 (m, 2H, C_q__uino_). 

^13^C-NMR (101 MHz, D_2_O): δ 27.21 (CH_2_), 31.81 (CH_2_), 36.86 (SCH), 63.72 (NCH_2_), 122.83 (C_quino_), 126.27 (C_quino_), 128.26 (C_quino_), 130.55 (C_quino_), 131.91 (C_quino_), 134.43 (C_quino_), 134.50 (C_quino_), 150.26 (C_quino_), 150.40 (C_quino_), 177.83 (COOH). 

Anal. Calcd for C_14_H_14_ClNO_2_S: C 56.85, H 4.77, N 4.74, Cl 11.99, S 10.84. Found: C 56.97, H 4.91, N 4.96, Cl 12.35, S 11.21.

*2-(3-Carboxypropyl)-2H,3H-[1,4]thiazino[2,3,4-ij]quinolin-4-ium chloride* (**5**). A solution of sulfuryl chloride (0.087 g, 0.64 mmol) in chloroform (10 mL) was added dropwise to a solution of di(8-quinolinyl) disulfide (0.206 g, 0.64 mmol) in chloroform (10 mL), and the mixture was stirred for 10 min at room temperature. A solution of hexenoic acid (0.147 g, 1.28 mmol) in chloroform (10 mL) was added dropwise, and the reaction mixture stirred for 1 h at room temperature and 8 h at reflux temperature. After cooling in the refrigerator, the formed precipitate was filtered off and dried in a vacuum, producing the product (0.286 g, 72% yield) as a yellow powder, mp 161–162 °C. 

^1^H-NMR (400 MHz, D_2_O): δ 1.68–1.86 (m, 4H, CH_2_), 2.36 (t, *J* = 6.7 Hz, 2H, CH_2_), 3.70–3.77 (m, 1H, SCH), 4.97 (dd, *J* = 14.2, 7.8 Hz, 1H, NCH_2_), 5.26 (d, *J* = 14.2 Hz, 1H, NCH_2_), 7.69–7.73 (m, 1H, C_q__uino_), 7.88–7.90 (m, 1H, C_q__uino_), 7.95–7.99 (m, 2H, C_q__uino_), 9.02–9.09 (m, 2H, C_q__uino_). 

^13^C-NMR (101 MHz, D_2_O): δ 21.41 (CH_2_), 30.36 (CH_2_), 33.06 (CH_2_), 36.27 (SCH), 62.91 (NCH_2_), 121.78 (C_quino_), 125.90 (C_quino_), 127.18 (C_quino_), 129.60 (C_quino_), 131.06 (C_quino_), 133.43 (C_q__uino_), 133.67 (C_quino_), 149.16 (C_quino_), 149.25 (C_q__uino_), 178.03 (COOH). 

Anal. Calcd for C_15_H_16_ClNO_2_S: C 58.15, H 5.21, N 4.52, Cl 11.44, S 10.35. Found: C 58.73, H 5.61, N 4.69, Cl 11.89, S 10.91.

*2-(Chloromethyl)-2H,3H-[1,4]thiazino[2,3,4-ij]quinolin-4-ium chloride* (**6**). A solution of sulfuryl chloride (0.065 g, 0.48 mmol) in chloroform (10 mL) was added dropwise to a solution of di(8-quinolinyl) disulfide (0.154 g, 0.48 mmol) in chloroform (10 mL), and the mixture was stirred for 10 min at room temperature. A solution of allyl chloride (0.073 g, 0.96 mmol) in chloroform (10 mL) was added dropwise, and the reaction mixture stirred for 1 h at room temperature and 8 h at reflux temperature. The mixture was filtered and the solvent was removed by rotary evaporator. The residue was dried in a vacuum, producing the product (0.260 g, 98% yield) as a yellow powder, mp 138–140 °C. 

^1^H-NMR (400 MHz, D_2_O): δ 3.66–3.71 (m, 1H, CH_2_), 4.03 (dd, *J* = 11.7, 5.6 Hz, 1H, CH_2_), 4.20 (s, 1H, SCH), 5.34 (d, *J* = 14.3 Hz, 1H, NCH_2_), 5.48 (dd, *J* = 14.3, 4.7 Hz, 1H, NCH_2_), 7.84–7.88 (m, 1H, C_q__uino_), 8.03–8.12 (m, 3H, C_q__uino_), 9.12–9.20 (m, 2H, C_q__uino_).

^13^C-NMR (101 MHz, D_2_O): δ 36.56 (CH_2_), 42.69 (SCH), 58.84 (NCH_2_), 121.86 (C_quino_), 124.05 (C_q__uino_), 127.31 (C_quino_), 129.57 (C_quino_), 130.95 (C_quino_), 131.73 (C_q__uino_), 133.43 (C_quino_), 149.40 (C_quino_), 149.60 (C_quino_). 

Anal. Calcd for C_12_H_11_Cl_2_NS: C 52.95, H 4.07, N 5.15, Cl 26.05, S 11.78. Found: C 53.13, H 4.17, N 5.39, Cl 26.46, S 12.21.

*2-(Bromomethyl)-2H,3H-[1,4]thiazino[2,3,4-ij]quinolin-4-ium chloride* (**7**). A solution of sulfuryl chloride (0.082 g, 0.60 mmol) in chloroform (10 mL) was added dropwise to a solution of di(8-quinolinyl) disulfide (0.194 g, 0.60 mmol) in chloroform (10 mL), and the mixture was stirred for 10 min at room temperature. A solution of allyl bromide (0.147 g, 1.2 mmol) in chloroform (10 mL) was added dropwise, and the reaction mixture stirred for 1 h at room temperature and 8 h at reflux temperature and 16 h at room temperature. The mixture was filtered and the solvent was removed by rotary evaporator. The residue was dried in a vacuum, producing the product (0.342 g, 90% yield) as a yellow powder, mp 162–164 °C. 

^1^H-NMR (400 MHz, D_2_O): δ 3.70–3.76 (m, 1H, CH_2_), 4.04–4.09 (m, 1H, CH_2_), 4.25 (s, 1H, SCH), 5.38 (d, *J* = 14.7 Hz, 1H, NCH_2_), 5.38 (d, *J* = 14.7 Hz, 1H, NCH_2_), 7.90–7.92 (m, 1H, C_q__uino_), 8.09 (s, 2H, C_q__uino_), 8.14–8.16 (m, 1H, C_q__uino_), 9.16–9.18 (m, 1H, C_q__uino_), 9.22 (s, 1H, C_q__uino_). 

^13^C-NMR (101 MHz, D_2_O): δ 36.53 (CH_2_), 42.65 (SCH), 58.81 (NCH_2_), 121.83 (C_quino_), 124.34 (C_q__uino_), 127.24 (C_quino_), 129.51 (C_quino_), 130.89 (C_q__uino_), 132.62 (C_q__uino_), 133.35 (C_quino_), 149.34 (C_quino_), 149.58 (C_quino_). 

Anal. Calcd for C_12_H_11_BrClNS: C 45.52, H 3.50, N 4.42, Br 25.23, Cl 11.20, S 10.13. Found: C 45.83, H 3.71, N 4.59, Br 25.64, Cl 11.56, S 10.69.

*2-(Cyanomethyl)-2H,3H-[1,4]thiazino[2,3,4-ij]quinolin-4-ium chloride* (**8**). A solution of sulfuryl chloride (0.059 g, 0.44 mmol) in chloroform (10 mL) was added dropwise to a solution of di(8-quinolinyl) disulfide (0.140 g, 0.44 mmol) in chloroform (10 mL), and the mixture was stirred for 10 min at room temperature. A solution of allyl cyanide (0.059 g, 0.88 mmol) in chloroform (10 mL) was added dropwise, and the reaction mixture stirred for 1 h at room temperature and 8 h at reflux temperature. After cooling in the refrigerator, the formed precipitate was filtered off and dried in a vacuum, producing the product (0.223 g, 96% yield) as a yellow powder, mp 183–185 °C. 

^1^H-NMR (400 MHz, (CD_3_)_2_CO): δ 2.97 (qd, *J* = 17.5, 7.0 Hz, 1H, CH_2_), 4.18 (dd, *J* = 12.8, 6.7 Hz, 1H, SCH), 5.11 (dd, *J* = 14.3, 6.7 Hz, 1H, NCH_2_), 5.48 (d, *J* = 14.3 Hz, 1H, NCH_2_), 7.71–7.75 (m, 1H, C_q__uino_), 7.88–8.00 (m, 3H, C_q__uino_), 8.99–9.02 (m, 2H, C_q__uino_). 

^13^C-NMR (101 MHz, (CD_3_)_2_CO): δ 20.65 (CH_2_), 31.61 (SCH), 60.67 (NCH_2_), 117.26 (CN), 121.58 (C_quino_), 123.30 (C_quino_), 125.36 (C_quino_), 127.45 (C_quino_), 129.43 (C_quino_), 130.64 (C_quino_), 133.53 (C_quino_), 149.33 (C_q__uino_), 149.45 (C_quino_). 

Anal. Calcd for C_13_H_11_ClN_2_S: C 59.42, H 4.22, N 10.66, Cl 13.49, S 12.20. Found: C 59.83, H 4.47, N 10.99, Cl 14.00, S 12.74.

### 3.3. Synthesis of Compounds ***9**–**13***

*3-(1H-Imidazol-1-yl)-2H,3H-[1,4]thiazino[2,3,4-ij]quinolin-4-ium chloride* (**9**). A solution of sulfuryl chloride (0.081 g, 0.60 mmol) in methylene chloride (10 mL) was added dropwise to a solution of di(8-quinolinyl) disulfide (0.192 g, 0.60 mmol) in methylene chloride (10 mL), and the mixture was stirred for 10 min at room temperature. A solution of 1-vinylimidazole (0.113 g, 1.2 mmol) in methylene chloride (10 mL) was added dropwise, and the reaction mixture was stirred for 48 h at room temperature. The formed precipitate was filtered off, washed with cold hexane and dried in a vacuum, producing the product (0.263 g, 75% yield) as a dark yellow powder, mp 127–129 °C. 

^1^H-NMR (400 MHz, methanol-*d*_4_): δ 3.69 (dd, *J* = 14.2, 3.9 Hz, 1H, SCH_2_), 3.82 (dd, *J* = 14.2, 1.4 Hz, 1H, SCH_2_), 6.41 (s, 1H, NCH), 7.25 (s, 2H, CH=CH), 7.87 (t, *J* = 7.9 Hz, 1H, NCHN), 8.06–8.18 (m, 4H, C_q__uino_), 9.27–9.29 (m, 1H, C_q__uino_), 9.51–9.52 (m, 1H, C_q__uino_). 

^13^C-NMR (101 MHz, methanol-*d_4_*): δ 29.45 (t, *J*_C–H_ 145.7 Hz, SCH_2_), 94.35 (d, *J*_C–H_ 168.6 Hz, NCH), 121.41 (CH=CH), 122.50 (C_q__uino_), 128.31 (C_q__uino_), 128.49 (C_q__uino_), 130.65 (C_q__uino_), 133.19 (C_q__uino_), 133.62 (C_q__uino_), 134.00 (C_q__uino_), 135.89 (C_q__uino_), 150.05 (C_q__uino_), 152.11 (N=CHN). 

Anal. Calcd for C_14_H_12_N_3_ClS: C 58.03, H 4.17, Cl 12.23, N 14.50, S 11.07. Found: C 55.94, H 4.52, Cl 12.69, N 5.23, S 11.51.

*3-(2-Oxopyrrolidin-1-yl)-2H,3H-[1,4]thiazino[2,3,4-ij]quinolin-4-ium perchlorate* (**10**). A solution of sulfuryl chloride (0.107 g, 0.79 mmol) in methylene chloride (10 mL) was added dropwise to a solution of di(8-quinolinyl) disulfide (0.254 g, 0.79 mmol) in methylene chloride (10 mL), and the mixture was stirred for 10 min at room temperature. Anhydrous KClO_4_ (0.219 g, 1.58 mmol) was added and the mixture was stirred for 10 min. A solution of *N*-vinylpyrrolidone (0.176 g, 1.58 mmol) in methylene chloride (10 mL) was added dropwise, and the reaction mixture was stirred for 24 h at room temperature. After filtration the solvent was removed by rotary evaporator. The residue was recrystallized from methanol/ether 1:1 and dried in a vacuum, producing the product (0.41 g, 70% yield) as a orange powder, mp 149–150 °C. 

^1^H-NMR (400 MHz, D_2_O): δ 2.06–2.25 (m, 2H, CH_2_), 2.61–2.67 (m, 2H, CH_2_), 3.37–3.42 (m, 1H, SCH_2_), 3.80–3.88 (m, 2H, CH_2_), 3.97–4.00 (m, 1H, SCH_2_), 7.19 (s, 1H, NCH), 7.92–7.96 (m, 1H, C_q__uino_), 8.10–8.15 (m, 1H, C_q__uino_), 8.18–8.22 (m, 1H, C_q__uino_), 9.21–9.25 (m, 2H, C_q__uino_). 

^13^C-NMR (101 MHz, D_2_O): δ 18.24 (CH_2_), 26.61 (CH_2_), 30.73 (CH_2_), 45.32 (SCH_2_), 72.01 (NCH), 122.28 (C_quino_), 125.66 (C_quino_), 128.45 (C_quino_), 129.91 (C_quino_), 132.00 (C_quino_), 134.39 (C_quino_), 134.96 (C_q__uino_), 147.10 (C_quino_), 151.10 (C_quino_), 180.49 (C=O). 

Anal. Calcd for C_15_H_15_N_2_O_5_ClS: C 48.59, H 4.08, N 7.55, Cl 9.56, S 8.65. Found: C 48.69, H 4.21, N 7.75, Cl 9.70, S 8.91.

*3-Ethoxy-2H,3H-[1,4]thiazino[2,3,4-ij]quinolin-4-ium chloride* (**11**). A solution of sulfuryl chloride (0.045 g, 0.33 mmol) in methylene chloride (10 mL) was added dropwise to a solution of di(8-quinolinyl) disulfide (0.106 g, 0.48 mmol) in methylene chloride (5 mL), and the mixture was stirred for 10 min at room temperature. A solution of vinyl ethyl ether (0.048 g, 0.66 mmol) in methylene chloride (10 mL) was added dropwise, and the reaction mixture stirred for 20 h at room temperature. The solvent was removed by rotary evaporator and the residue was dried in a vacuum, producing the product (0.177 g, ~100% yield) as an orange oil. 

^1^H-NMR (400 MHz, D_2_O): δ 1.19 (t, *J* = 7.0 Hz, 3H, CH_3_), 3.66–3.74 (m, 2H, SCH_2_, OCH_2_), 3.81 (dd, *J* = 14.3, 1.9 Hz, 1H, SCH_2_), 4.00 (dd, *J* = 9.2, 7.0 Hz, 1H, OCH_2_), 6.52 (d, *J* = 1.9 Hz, 1H, NCH), 7.81–7.85 (m, 1H, C_q__uino_), 8.04–8.06 (m, 1H, C_q__uino_), 8.10–8.15 (m, 2H, C_q__uino_), 9.21–9.24 (m, 1H, C_q__uino_), 9.38–9.40 (m, 1H, C_q__uino_). 

^13^C-NMR (101 MHz, D_2_O): δ 13.81 (CH_3_), 28.51 (SCH_2_), 66.27 (OCH_2_), 91.48 (NCH), 121.24 (C_quino_), 125.52 (C_quino_), 127.44 (C_quino_),128.09 (C_q__uino_), 129.39 (C_quino_), 131.55 (C_quino_), 133.08 (C_quino_), 148.21 (C_quino_), 151.05 (C_quino_). 

Anal. Calcd for C_13_H_14_NClOS: C 58.31, H 5.27, N 5.23, Cl 13.24, S 11.97. Found: C 58.60, H 5.36, N 5.54, Cl 13.61, S 12.42. 

*3-Butoxy-2H,3H-[1,4]thiazino[2,3,4-ij]quinolin-4-ium chloride* (**12**). A solution of sulfuryl chloride (0.077 g, 0.57 mmol) in methylene chloride (10 mL) was added dropwise to a solution of di(8-quinolinyl) disulfide (0.183 g, 0.57 mmol) in methylene chloride (5 mL), and the mixture was stirred for 10 min at room temperature. A solution of vinyl butyl ether (0.114 g, 1.14 mmol) in methylene chloride (10 mL) was added dropwise, and the reaction mixture stirred for 24 h at room temperature. The solvent was removed by rotary evaporator and the residue was dried in a vacuum, producing the product (0.338 g, ~100% yield) as a light yellow powder, mp 139–140 °C. 

^1^H-NMR (400 MHz, D_2_O): δ 0.72 (t, *J* = 7.4 Hz, 3H, CH_3_), 1.16 (dt, *J* = 15.5, 7.5 Hz, 2H, CH_2_), 1.50 (s, 2H, CH_2_), 3.58 (d, *J* = 7.8 Hz, 1H, OCH_2_), 3.72 (d, *J* = 14.0 Hz, 1H, SCH_2_), 3.82 (d, *J* = 14.0 Hz, 1H, SCH_2_), 3.94 (d, *J* = 8.8 Hz, 1H, OCH_2_), 6.49 (s, 1H, NCH), 7.87–7.91 (m, 1H, C_q__uino_), 8.07–8.16 (m, 3H, C_q__uino_), 9.22–9.24 (m, 1H, C_q__uino_), 9.34–9.36 (m, 1H, C_q__uino_). 

^13^C-NMR (101 MHz, D_2_O): δ 12.64 (CH_3_), 18.26 (CH_2_), 28.44 (CH_2_), 30.12 (SCH_2_), 69.95 (OCH_2_), 91.51 (NCH), 121.09 (C_quino_), 124.41 (C_q__uino_), 125.11 (C_q__uino_), 127.42 (C_quino_), 129.47 (C_quino_), 131.64 (C_quino_), 133.10 (C_quino_), 148.08 (C_quino_), 151.08 (C_quino_). 

Anal. Calcd for C_15_H_18_NClOS: C 60.90, H 6.13, N 4.73, Cl 11.98, S 10.84. Found: C 61.20, H 6.36, N 5.04, Cl 12.29, S 11.10.

*7aH,8H,9H,10aH-Furo[2′,3′:5,6][1,4]thiazino[2,3,4-ij]quinolin-11-ium perchlorate* (**13**). A solution of sulfuryl chloride (0.079 g, 0.58 mmol) in methylene chloride (10 mL) was added dropwise to a solution of di(8-quinolinyl) disulfide (0.187 g, 0.58 mmol) in methylene chloride (10 mL), and the mixture was stirred for 10 min at room temperature. Anhydrous KClO_4_ (0.162 g, 1.17 mmol) was added and the mixture was stirred for 10 min. A solution of 2,3-dihydrofuran (0.082 g, 1.17 mmol) in methylene chloride (10 mL) was added dropwise, and the reaction mixture was stirred for 48 h at room temperature. After filtration the solvent was removed by rotary evaporator. The residue was recrystallized from methanol/ether 1:1 and dried in a vacuum, producing the product (0.278 g, 72% yield) as an orange powder, mp 219–220 °C. 

^1^H-NMR (400 MHz, DMSO-*d*_6_): δ 1.81–1.92 (m, 1H, CH_2_), 2.60–2.64 (m, 1H, CH_2_), 4.10–4.15 (m, 1H, SCH), 4.22–4.33 (m, 2H, CH_2_O), 6.52 (d, *J* = 4.7 Hz, 1H, NCH), 7.82–7.86 (m, 1H, C_q__uino_), 8.02–8.04 (m, 1H, C_q__uino_), 8.15–8.20 (m, 1H, C_q__uino_), 9.19–9.21 (m, 1H, C_q__uino_), 9.57–9.58 (1H, C_q__uino_). 

^13^C-NMR (101 MHz, DMSO-*d*_6_): δ 28.50 (CH_2_), 37.36 (SCH), 68.90 (CH_2_O), 91.62 (NCH), 121.84 (C_q__uino_), 122.15 (C_q__uino_), 127.44 (C_q__uino_), 129.19 (C_q__uino_), 130.24 (C_q__uino_), 132.91 (C_q__uino_), 133.40 (C_q__uino_), 146.62 (C_q__uino_), 149.24 (C_q__uino_). 

Anal. Calcd for C_13_H_12_NClO_5_S: C 47.35, H 3.67, N 4.25, Cl 10.75, S 9.72. Found: C 47.82, H 3.52, N 4.75, Cl 11.25, S 10.08.

## 4. Conclusions

Unsaturated heteroatom (4-pentenoic and 5-hexenoic acids, allyl chloride and bromide, allyl cyanate, ethyl vinyl and butyl vinyl ethers) and heterocyclic (*N*-vinyl pyrrolidin-2-one, 1-vinylimidazole and 2,3-dihydrofuran) compounds were used for the efficient regioselective synthesis of a novel family of [1,4]thiazino[2,3,4-*ij*]quinolin-4-ium derivatives by annulation reactions with 8-quinolinesulfenyl chloride.

The reactions with 4-pentenoic and 5-hexenoic acids, allylchloride, allylbromide and allyl cyanate included the electrophilic addition of the sulfur atom of sulfenyl chloride to the α-carbon atom of the vinyl group (“anti-Markovnikov direction”), while the annulation reactions with *N*-vinyl pyrrolidin-2-one, 1-vinylimidazole, 2,3-dihydrofuran, ethyl vinyl and butyl vinyl ethers proceeded with the attachment of the sulfur atom to the β-carbon atom of the vinyl group (“Markovnikov direction”). We presume that in the latter case the reactions proceed via linear intermediates (Scheme 7) which are stabilized by the nitrogen or oxygen atom. In the case of the anti-Markovnikov direction of the reactions, there are no heteroatoms adjacent to the double bond which could stabilize the intermediates, and the reactions take place via thiiranium intermediates.

The antibacterial activity of novel water-soluble compounds against *E. durans*, *B. subtilis* and *E. coli* was evaluated and the compounds with high antibacterial activity have been found (Table 1). Compound **5,** with its longer carbon chain, exhibited considerably higher activity against gram-positive *E. durans* and *B. subtilis* and was superior to antibiotic gentamicin in this respect. Bromo-containing compound **7** was 40 times more effective than its chlorine analogue **6** against *E. durans*. A comparison of compounds **11**–**13** revealed that products **11** and **13** (obtained from ethyl vinyl ether and 2,3-dihydrofuran) displayed superior activity compared to gentamicin against gram-positive bacteria. The highest activity was shown by product **10** (obtained from *N*-vinyl pyrrolidin-2-one), which significantly exceeded the activity of gentamicin and all obtained compounds against gram-positive bacteria and was more than a hundred times superior to this antibiotic against *B. subtilis*.

## Figures and Tables

**Table 1:** Evaluation of the antibacterial activity of compounds **1**, **4**–**13**.

No	Compound	Minimum Inhibitory Concentration (μg/mL)		
		*Enterococcus* *durans*	*Bacillus* *subtilis*	*Escherichia* *coli*
**1**	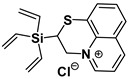			
		>1000	>1000	>1000
**4**	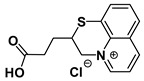			
		
		
		500	1000	>1000
**5**	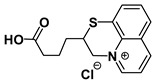			
		
		
		6.2	6.2	>1000
**6**	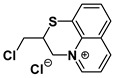			
		
		
		500	1000	31.2
**7**	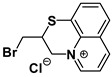			
		
		12.5	500	500
**8**	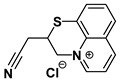			
		
		
		250	500	500
**9**	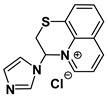			
		
		
		62.5	62.5	62.5
**10**	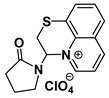			
		
		
		1.25	0.3	1000
**11**	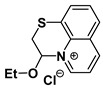			
		
		
		3.1	25	125
**12**	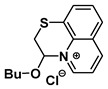			
		
		
		31.5	1000	125
**13**	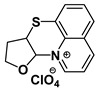			
		
		
		3.1	12.5	250
**Gentamicin** *				
		
		
		25	50	100

* Antibiotic gentamicin was used as a control compound.

## Data Availability

Not applicable.

## Supplementary Materials

The following are available online. Examples of NMR spectra of the obtained compounds.
