# Supplementary material for: A Novel Family of [1,4]Thiazino[2,3,4-ij]quinolin-4-ium Derivatives: Regioselective Synthesis Based on Unsaturated Heteroatom and Heterocyclic Compounds and Antibacterial Activity
Source: Molecules. 2021 Sep 14;26(18):5579. doi: 10.3390/molecules26185579 (PMC8472155; doi:10.3390/molecules26185579)
Supplement: Supplementary file 1 [file molecules-26-05579-s001.zip › molecules-1345158-supplementary.pdf]

A Novel Family of [1,4]thiazino[2,3,4-*ij*]quinolin-4-ium Derivatives: Regioselective Synthesis Based on Unsaturated Heteroatom and Heterocyclic Compounds and Antibacterial Activity

Vladimir A. Potapov*, Roman S. Ishigeev, Lyudmila A. Belovezhets and Svetlana V. Amosova

A. E. Favorsky Irkutsk Institute of Chemistry, Siberian Division of The Russian Academy of Sciences, 1 Favorsky Str., Irkutsk 664033, Russia; ishigeev@irioch.irk.ru (R.S.I.); belovezhets@irioch.irk.ru (L.A.B.); amosova@irioch.irk.ru (S.V.A.);

* Correspondence: v.a.potapov@mail.ru

Table of Contents

Experimental (General Information) 2

Examples of NMR Spectra 3-13

Experimental (General Information)

^{1}H (400.1 MHz) and ^{13}C (100.6 MHz) NMR spectra were recorded on a Bruker DPX-400 spectrometer (Bruker BioSpin GmbH, Rheinstetten, Germany) in 2-5% solution in D_2O. ^{1}H and ^{13}C chemical shifts (δ) are reported in parts per million (ppm), relative to tetramethylsilane (external) or to the residual solvent peaks of D_2O (δ = 4.79), acetone-$\boldsymbol{d}_6$ (δ = 2.05 and 29.84 ppm), methanol-$\boldsymbol{d}_4$ (δ = 3.31 and 49.0 ppm) and DMSO-$\boldsymbol{d}_6$ (δ = 2.50 and 39.52 ppm for ^{1}H and ^{13}C NMR, respectively). Elemental analysis was performed on a Thermo Scientific FLASH 2000 Organic Elemental Analyzer (Thermo Fisher Scientific Inc., Milan, Italy). Melting points were determined on a Kofler Hot-Stage Microscope PolyTherm A apparatus (Wagner & Munz GmbH, München, Germany). Absolute solvents were used in the reactions.

Examples of NMR spectra

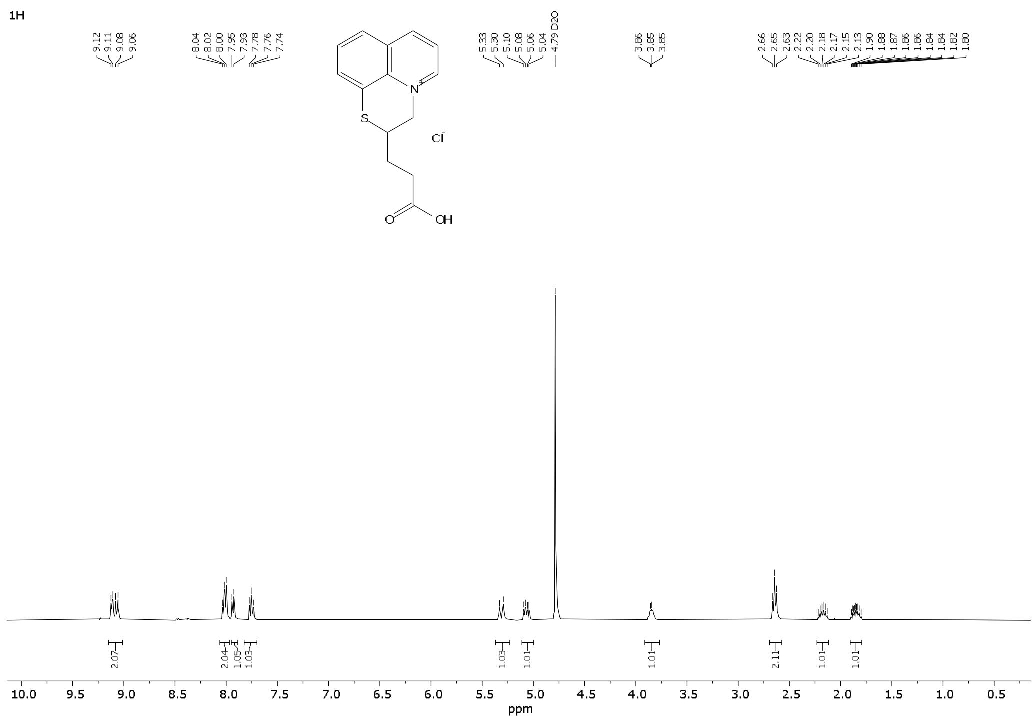

^{1}H-NMR (D_2O) spectrum of 2-(3-carboxyethyl)-2*H*,3*H*-[1,4]thiazino[2,3,4-*ij*]quinolin-4-ium chloride (4)

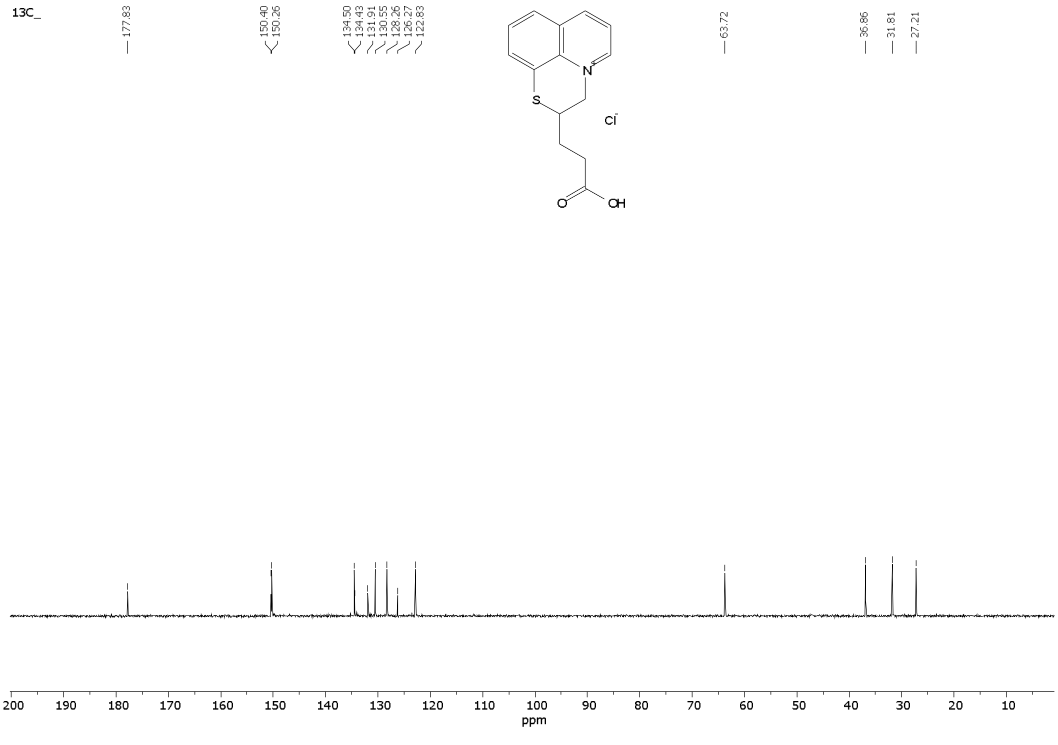

^{13}C-NMR (D_2O) spectrum of 2-(3-carboxyethyl)-2*H*,3*H*-[1,4]thiazino[2,3,4-*ij*]quinolin-4-ium chloride (4)

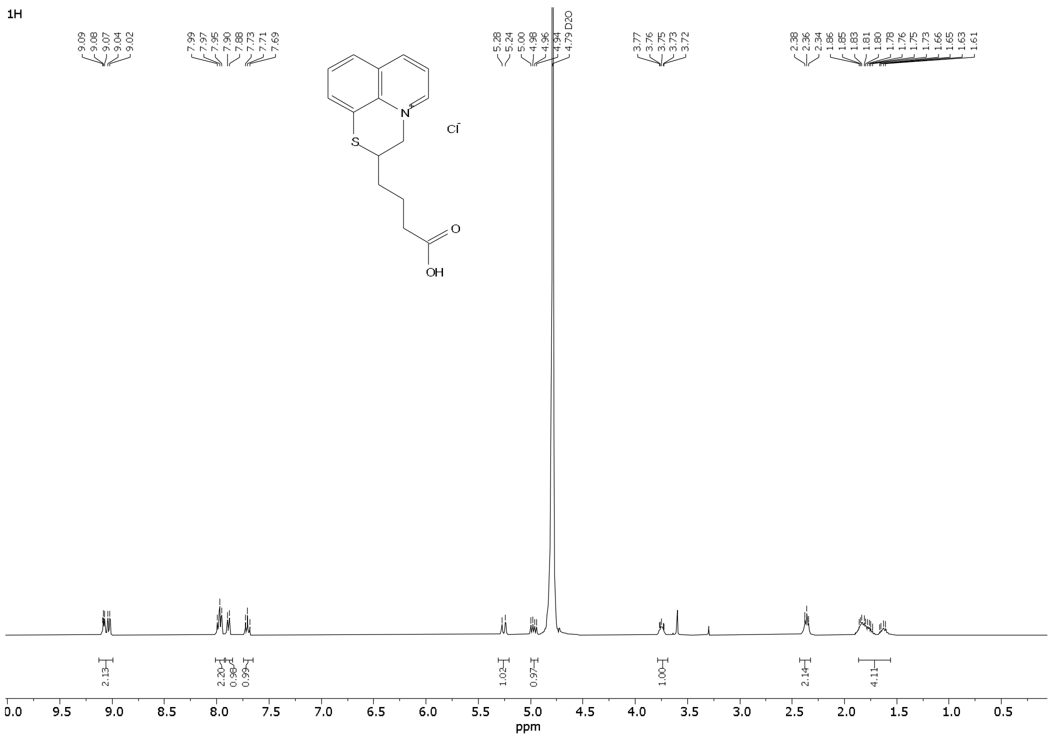

^{1}H-NMR (D_2O) spectrum of 2-(3-carboxypropyl)-2*H*,3*H*-[1,4]thiazino[2,3,4-*ij*]quinolin-4-ium chloride (5)

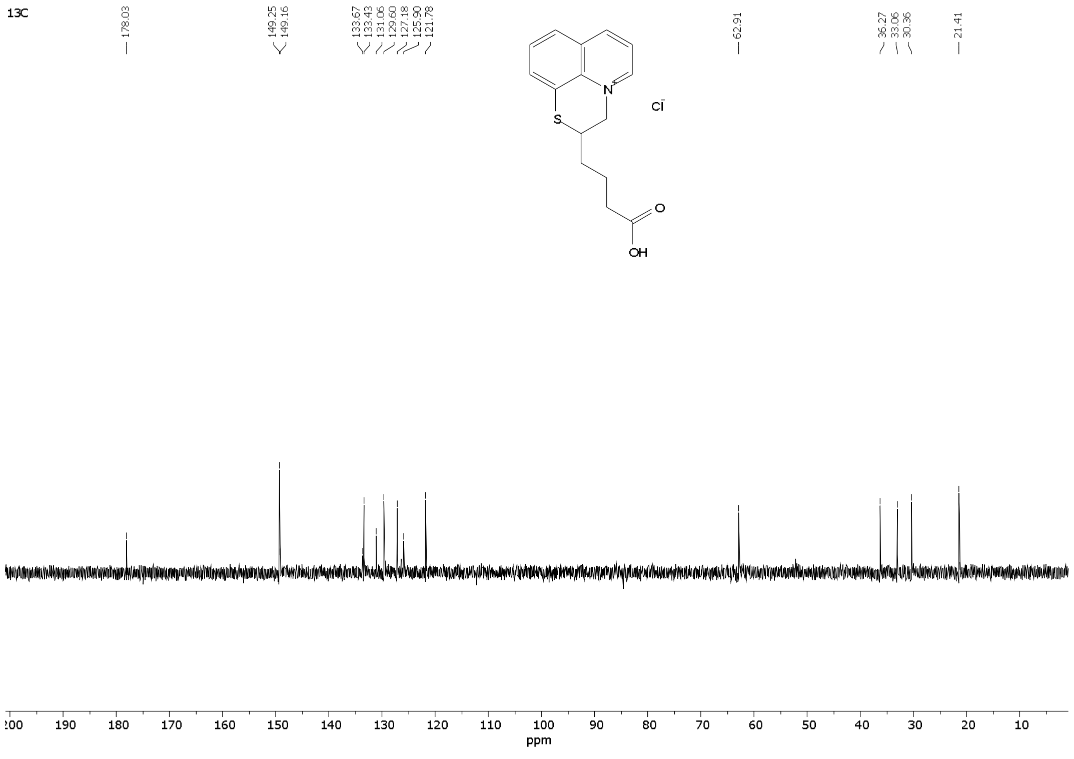

^{13}C-NMR (D_2O) spectrum of 2-(3-carboxypropyl)-2*H*,3*H*-[1,4]thiazino[2,3,4-*ij*]quinolin-4-ium chloride (5)

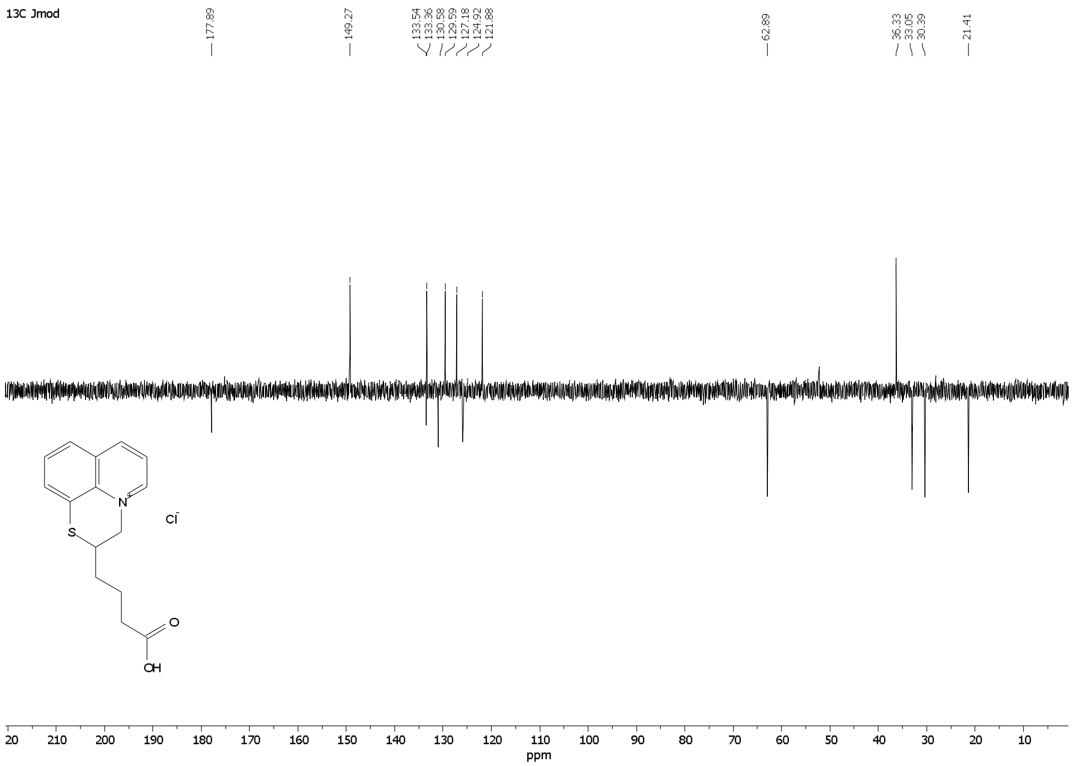

^{13}C-NMR (J_{mod}) spectrum (D_2O) of 2-(3-carboxypropyl)-*2H,3H*-[1,4]thiazino[2,3,4-*ij*]quinolin-4-ium chloride (5)

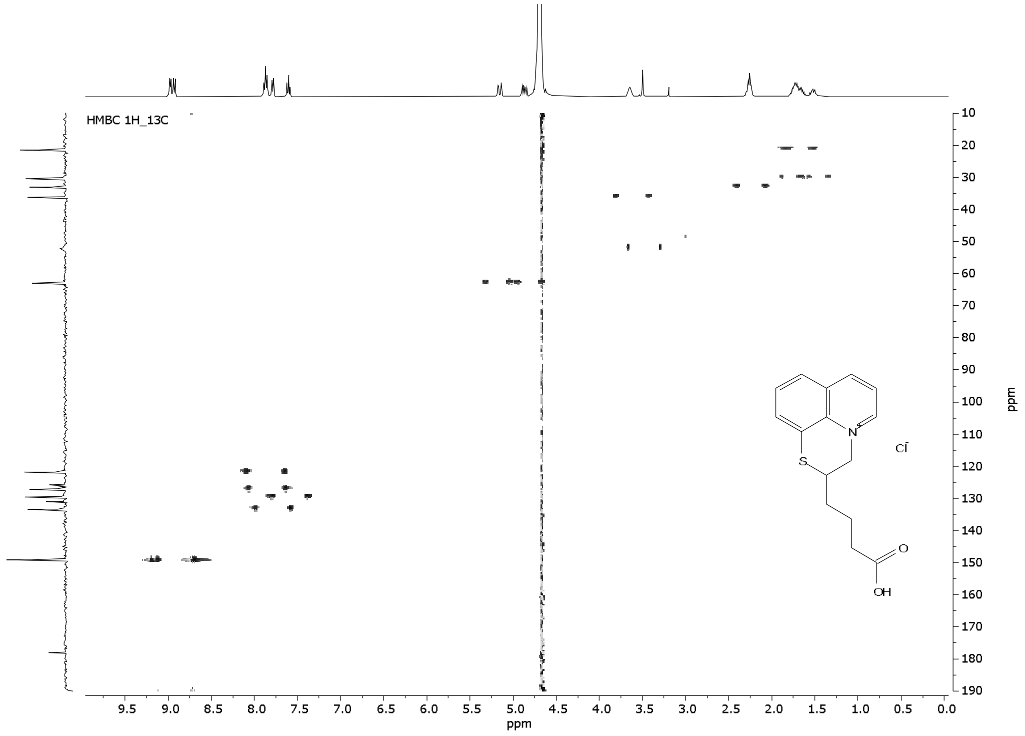

HMBC ^{1}H-^{13}C (D_2O) NMR spectrum of 2-(3-carboxypropyl)-2*H*,3*H*-[1,4]thiazino[2,3,4-*ij*]quinolin-4-ium chloride (5)

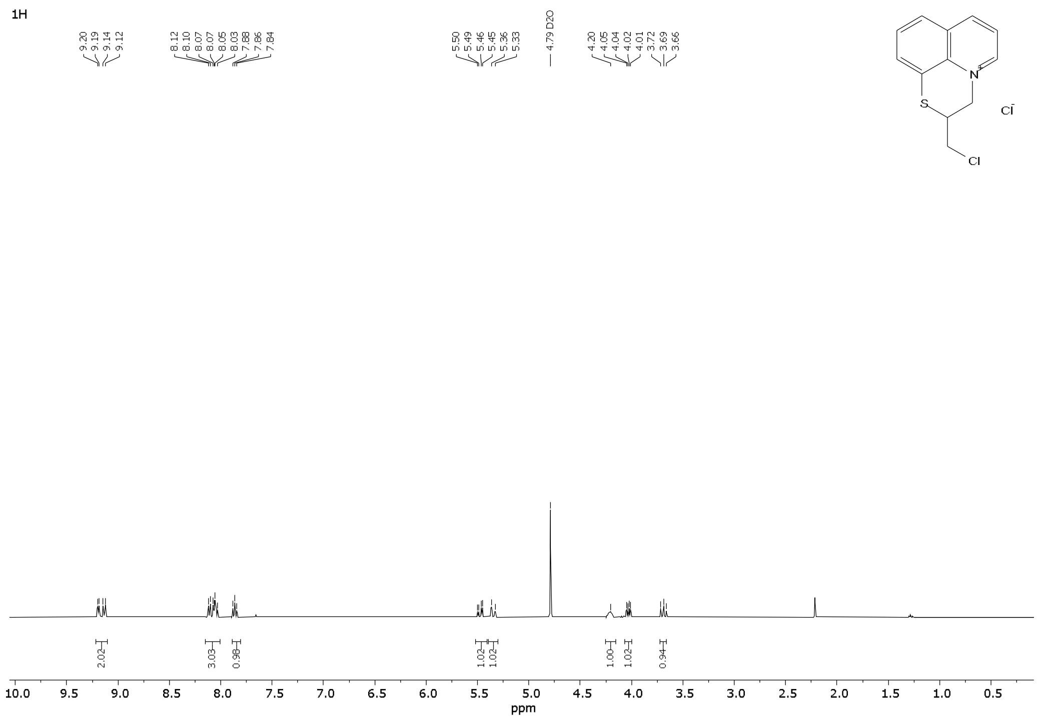

^{1}H-NMR (D_2O) spectrum of 2-(chloromethyl)-2*H*,3*H*-[1,4]thiazino[2,3,4-*ij*]quinolin-4-ium chloride (6)

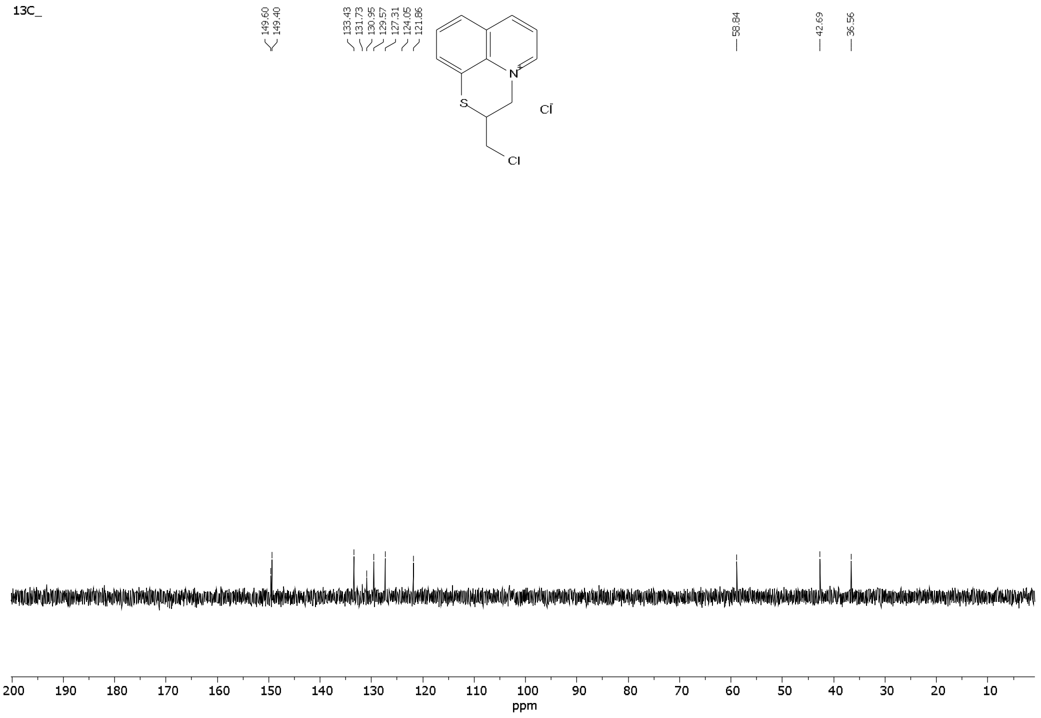

^{13}C-NMR (D_2O) spectrum of 2-(chloromethyl)-2*H*,3*H*-[1,4]thiazino[2,3,4-*ij*]quinolin-4-ium chloride (6)

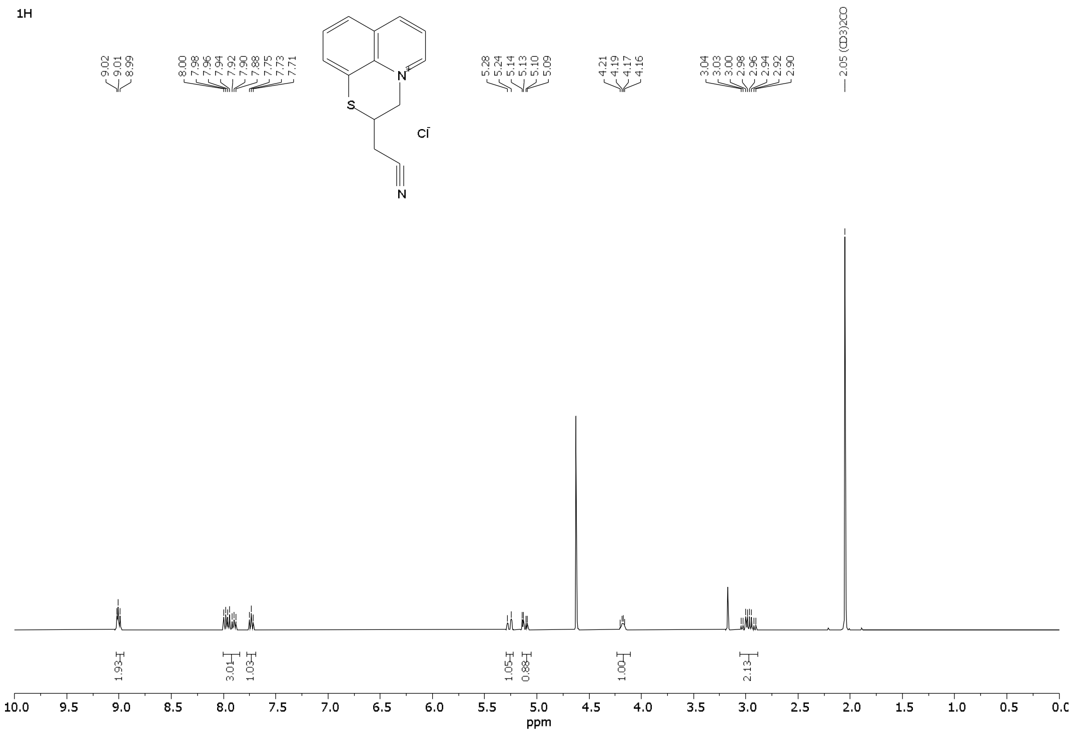

^{1}H-NMR (acetone-d_6) spectrum of 2-(cyanomethyl)-2*H*,3*H*-[1,4]thiazino[2,3,4-*ij*]quinolin-4-ium chloride (8)**

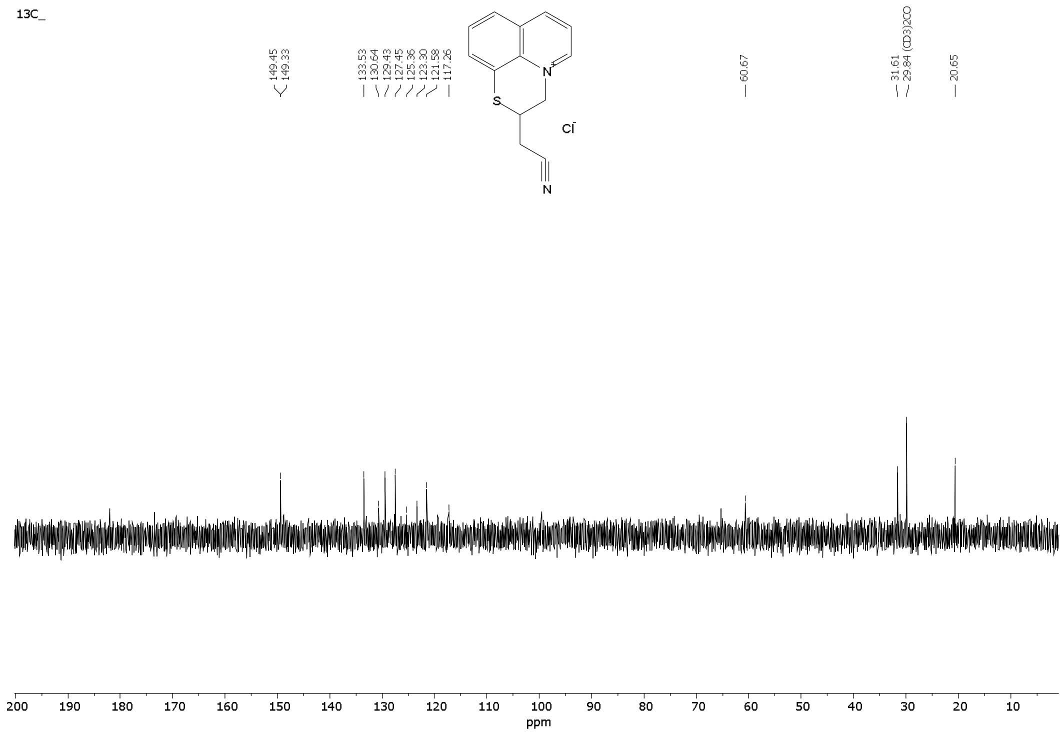

^{13}C-NMR (acetone-d_6) spectrum of 2-(cyanomethyl)-2*H*,3*H*-[1,4]thiazino[2,3,4-*ij*]quinolin-4-ium chloride (8)

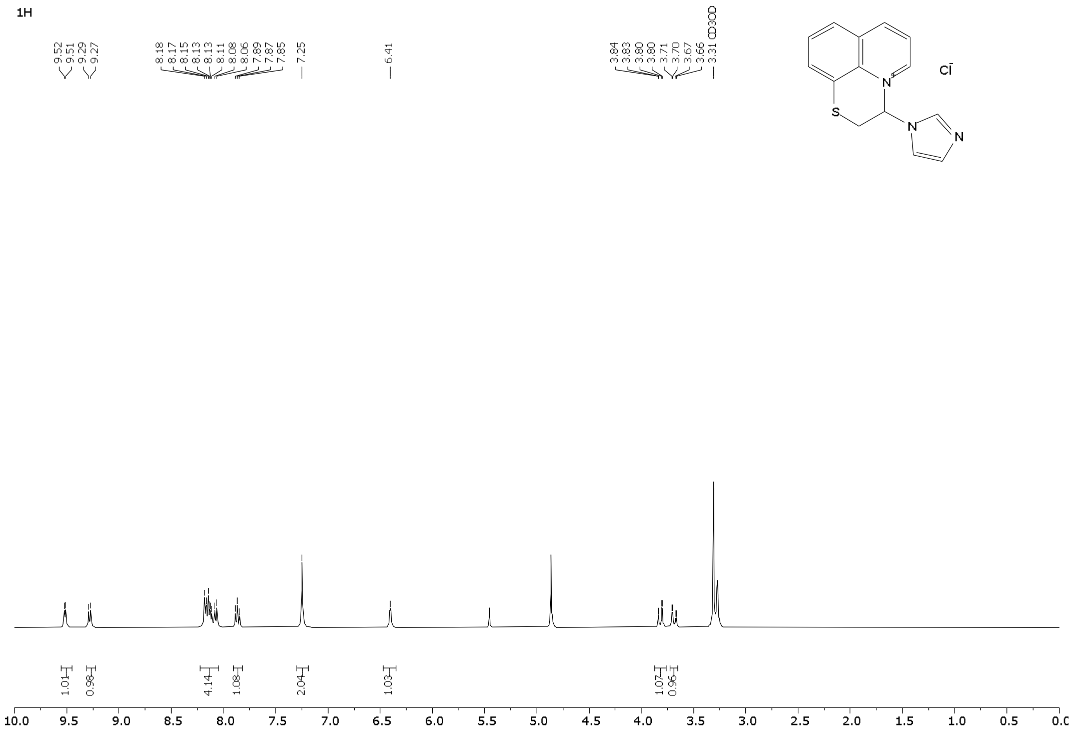

^{1}H-NMR (CD_3OD) spectrum of 3-(1*H*-imidazol-1-yl)-2*H*,3*H*-[1,4]thiazino[2,3,4-*ij*]quinolin-4-ium chloride (9)

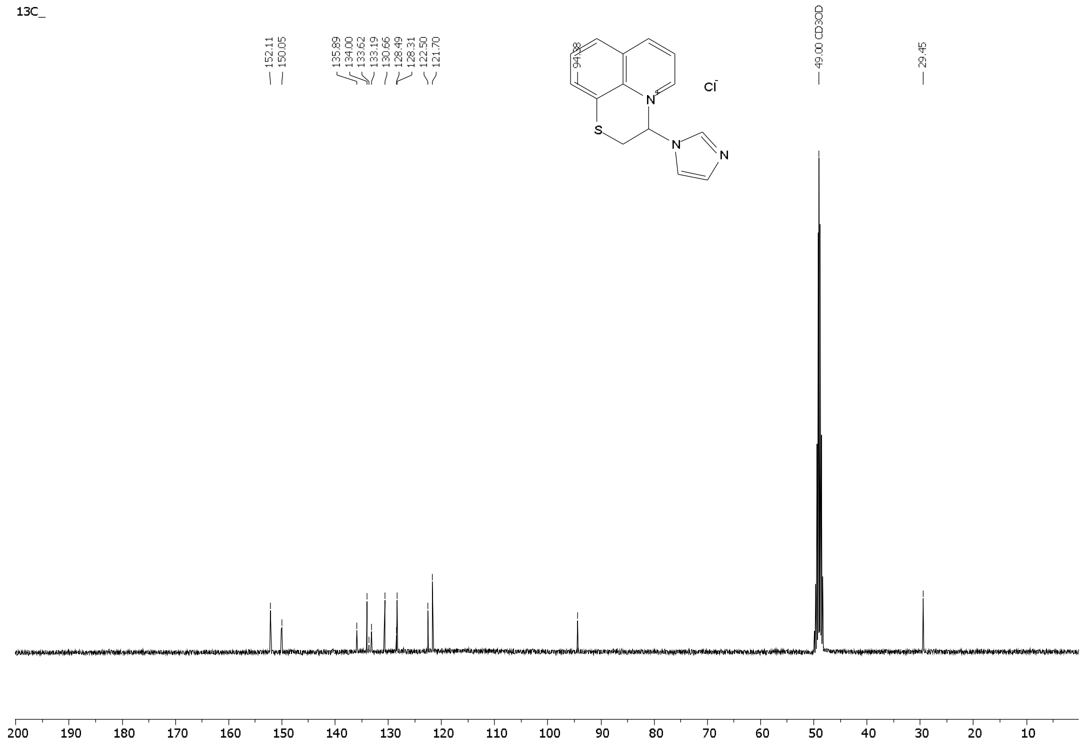

^{13}C-NMR (CD_3OD) spectrum of 3-(1*H*-imidazol-1-yl)-2*H*,3*H*-[1,4]thiazino[2,3,4-*ij*]quinolin-4-ium chloride (9)

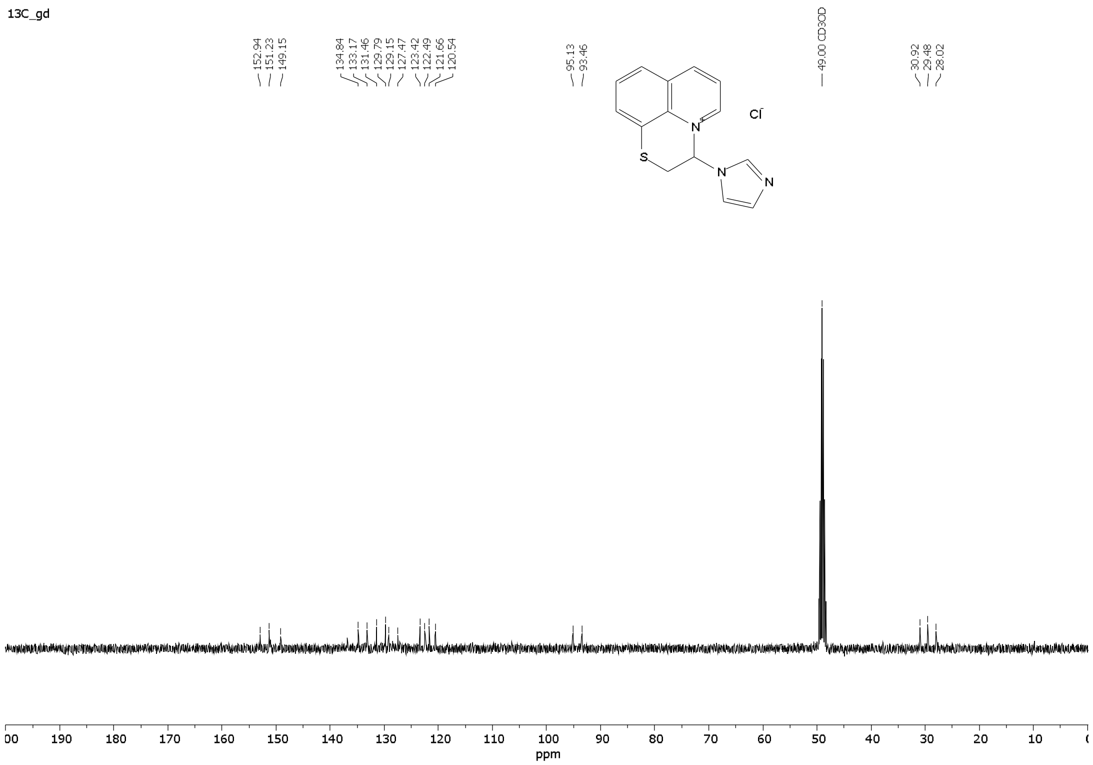

^{13}C-NMR (CD_3OD) proton-coupled spectrum of 3-(*1H*-imidazol-1-yl)-2*H*,3*H*-[1,4]thiazino[2,3,4-*ij*]quinolin-4-ium chloride (9)

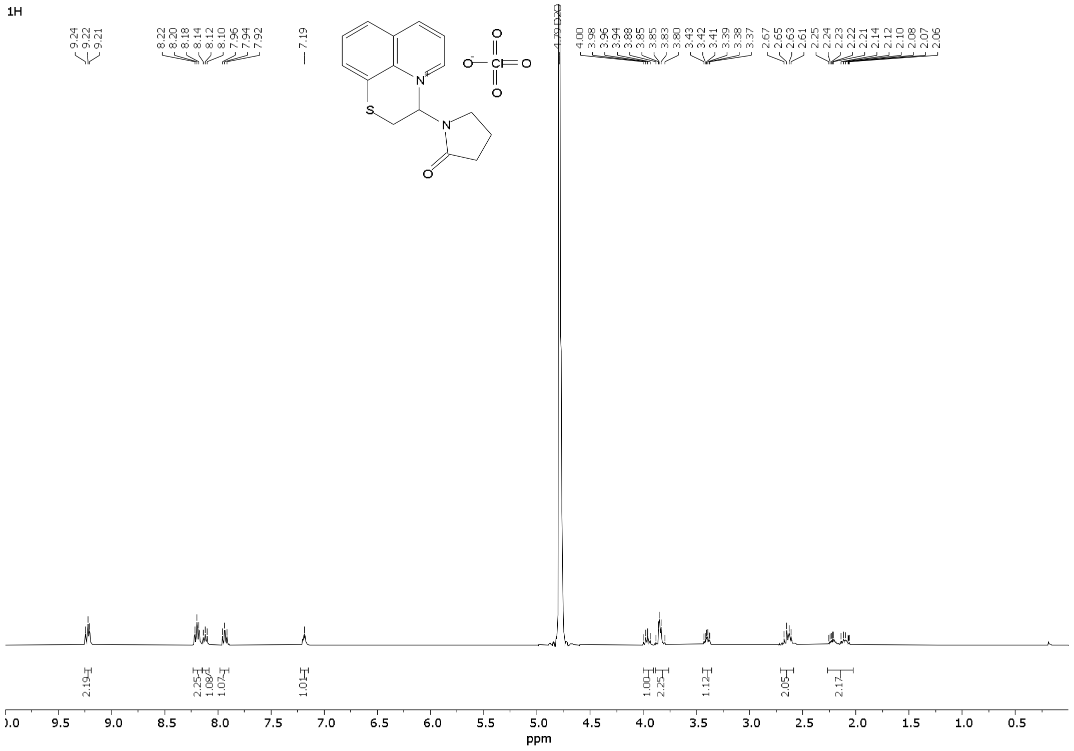

^{1}H-NMR (D_2O) spectrum of 3-(2-oxopyrrolidin-1-yl)-2*H*,3*H*-[1,4]thiazino[2,3,4-ij]quinolin-4-ium perchlorate (10)

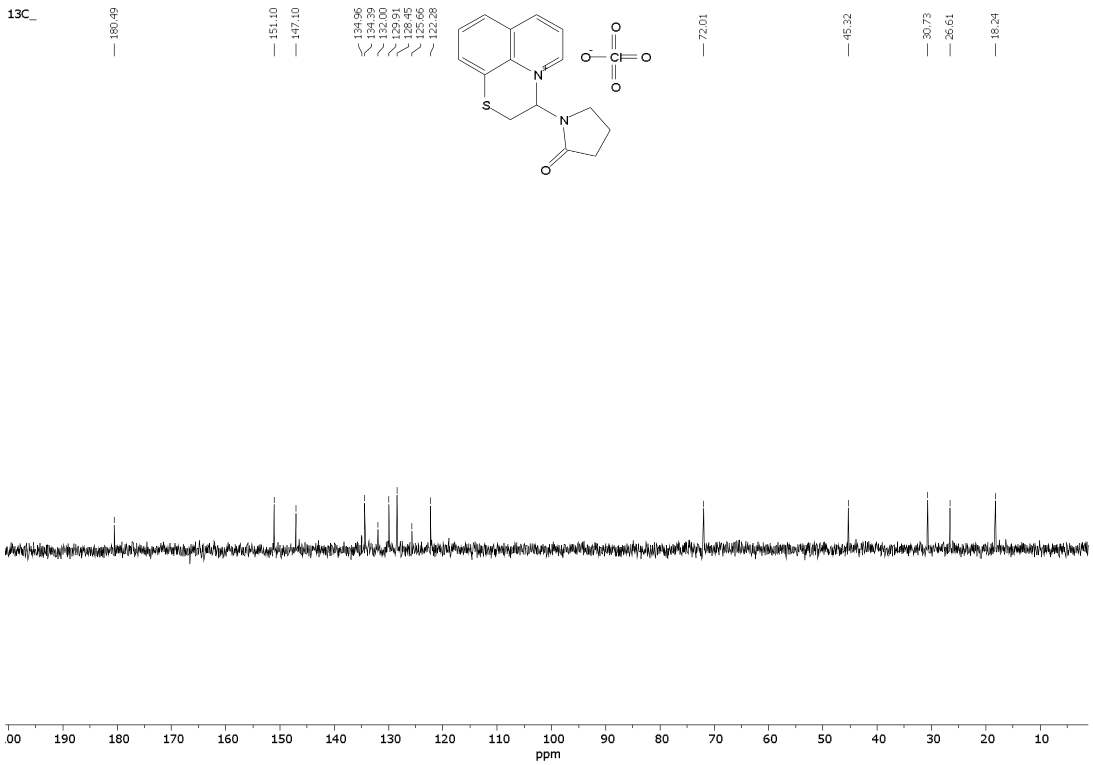

^{13}C-NMR (D_2O) spectrum of 3-(2-oxopyrrolidin-1-yl)-2*H*,3*H*-[1,4]thiazino[2,3,4-ij]quinolin-4-ium perchlorate (10)

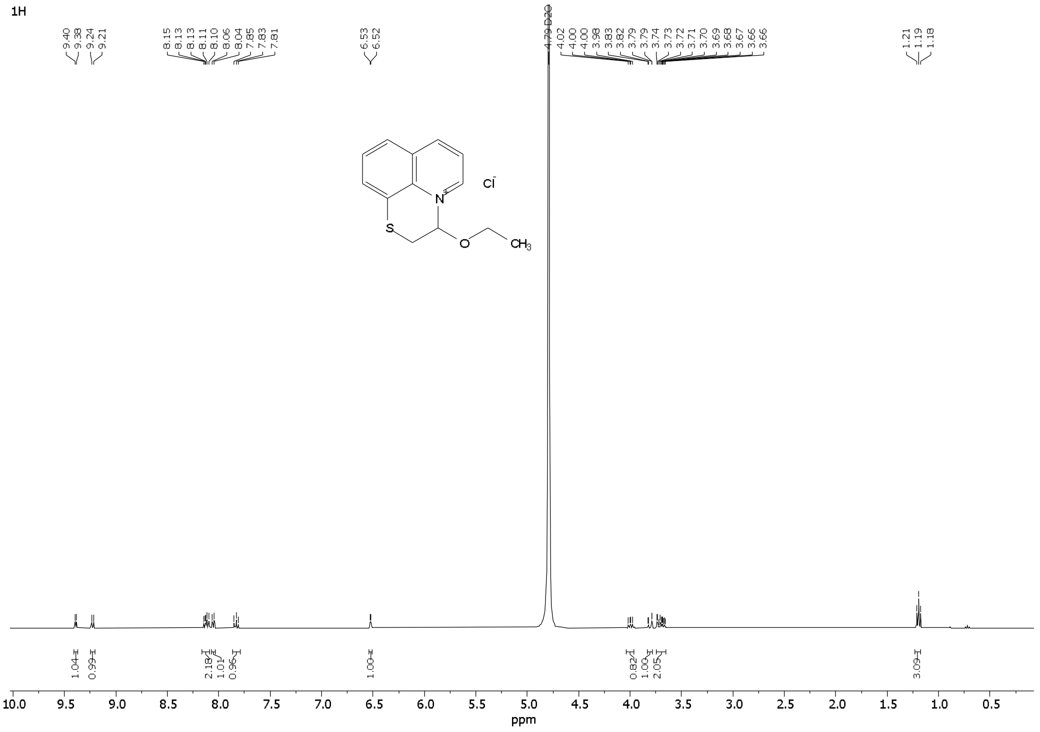

^{1}H-NMR (D_2O) spectrum of 3-ethoxy-2*H*,3*H*-[1,4]thiazino[2,3,4-*ij*]quinolin-4-ium chloride (11)

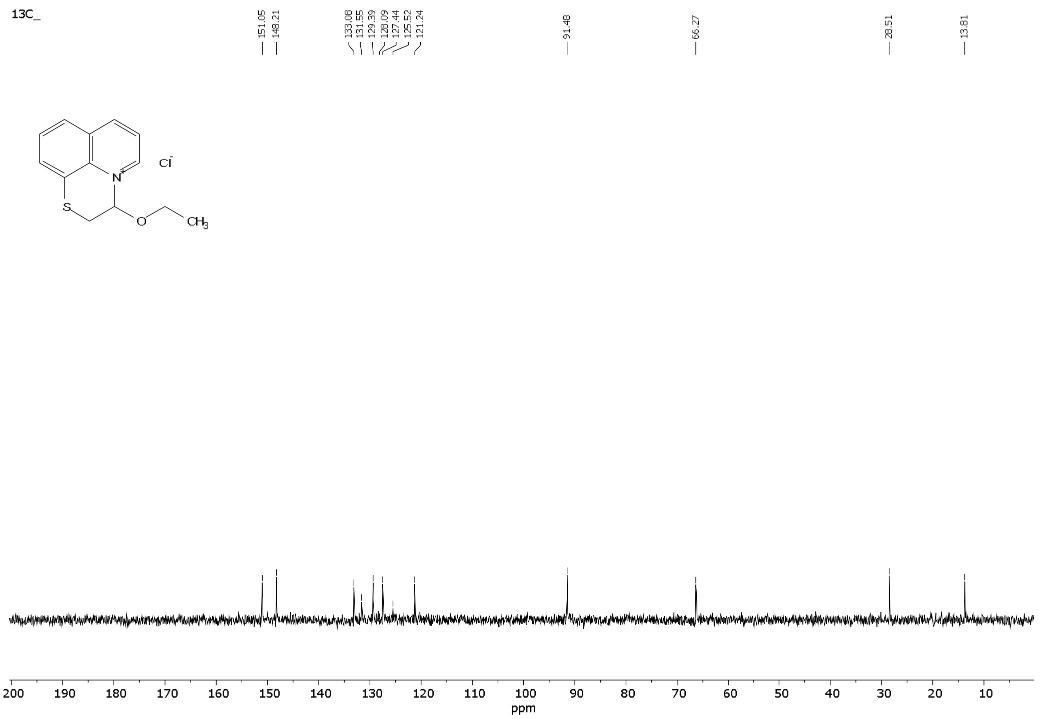

^{13}C-NMR (D_2O) spectrum of 3-ethoxy-2*H*,3*H*-[1,4]thiazino[2,3,4-*ij*]quinolin-4-ium chloride (11)

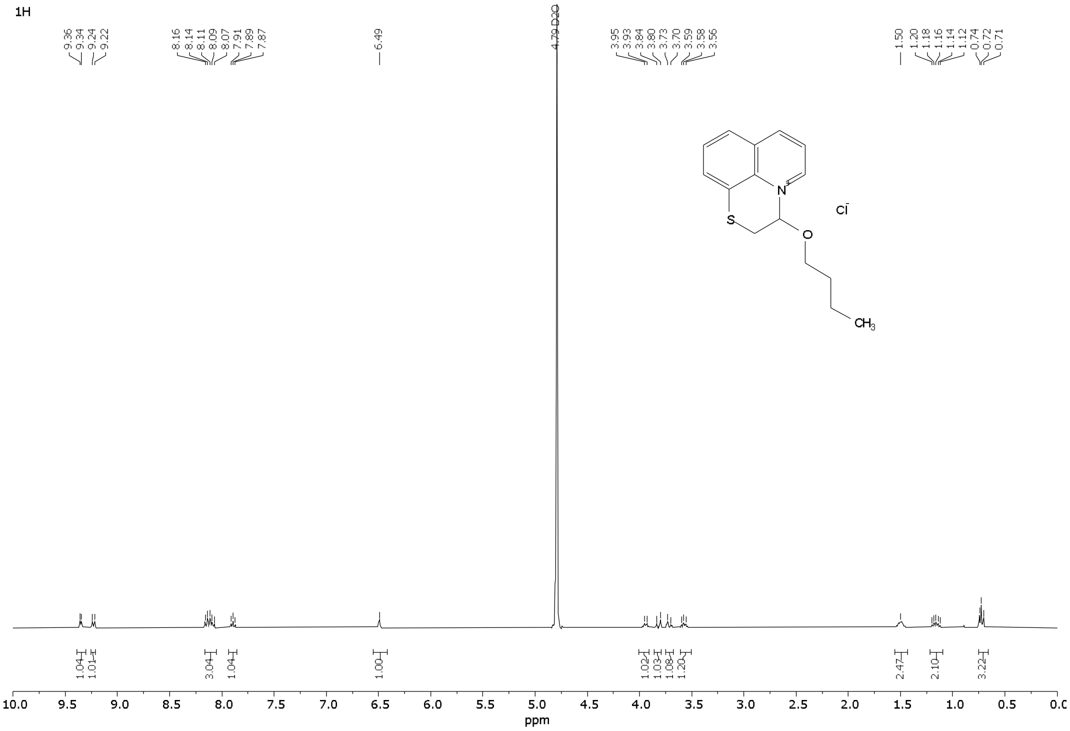

^{1}H-NMR (D_2O) spectrum of 3-butoxy-2*H*,3*H*-[1,4]thiazino[2,3,4-*ij*]quinolin-4-ium chloride (12)

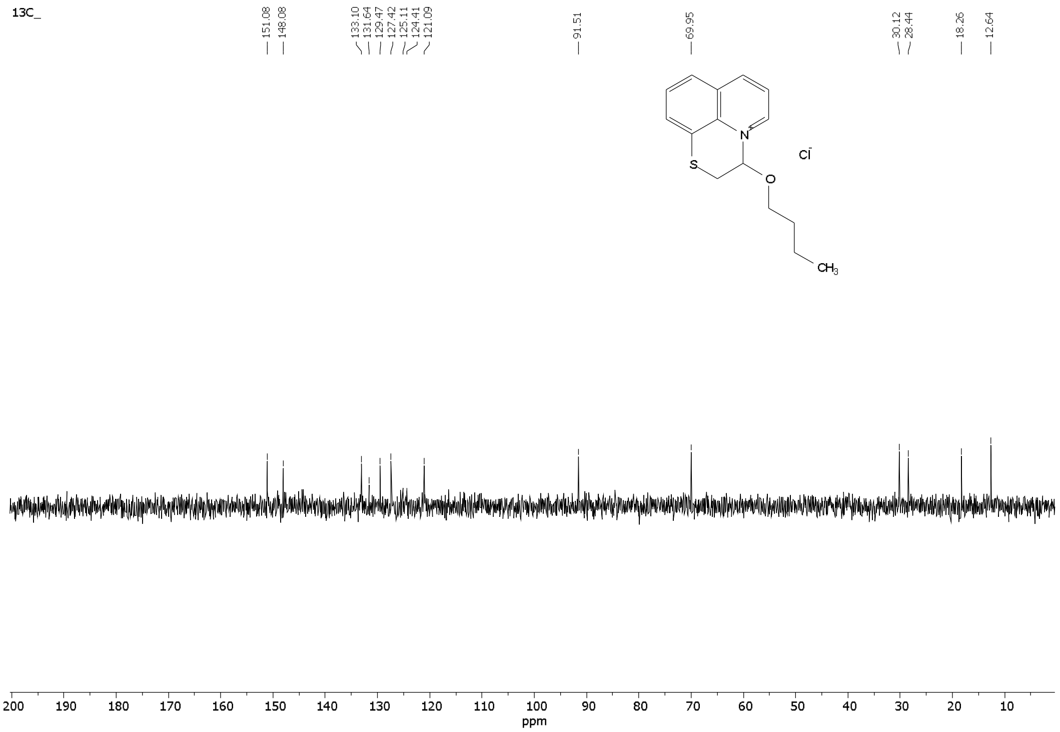

^{13}C-NMR (D_2O) spectrum of 3-butoxy-2*H*,3*H*-[1,4]thiazino[2,3,4-*ij*]quinolin-4-ium chloride (12)

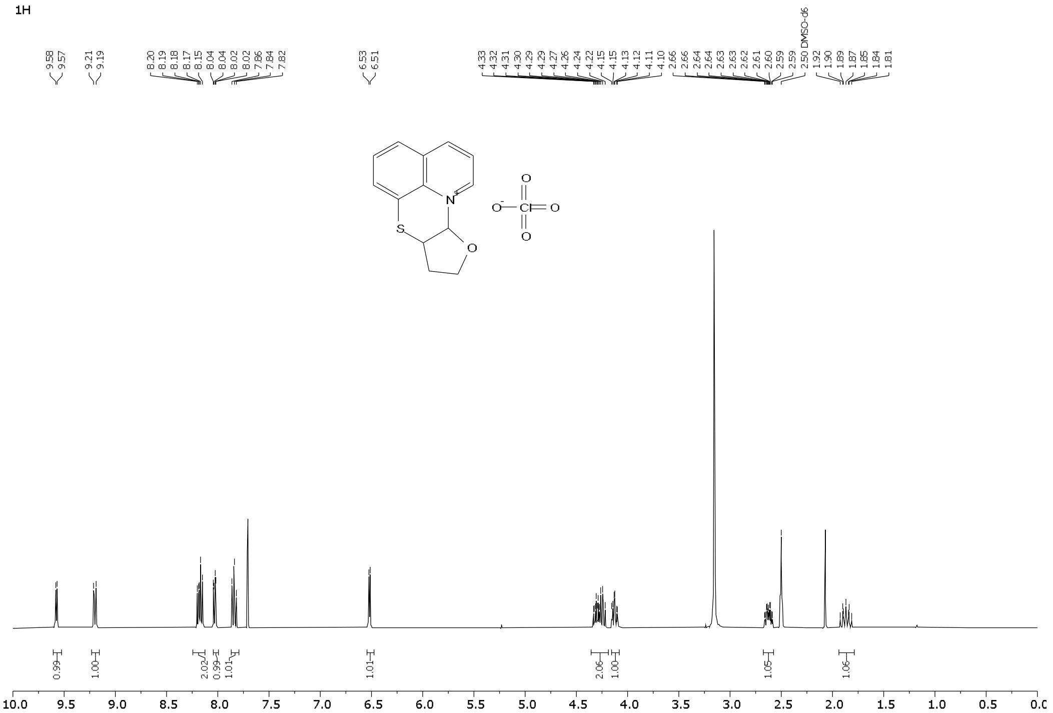

^{1}H-NMR (DMSO-d_6/$CDCl_3$, 1:1) spectrum of 7a*H*,8*H*,9*H*,10a*H*-furo[2’,3’:5,6][1,4]thiazino[2,3,4-*ij*]quinolin-11-ium perchlorate (13)

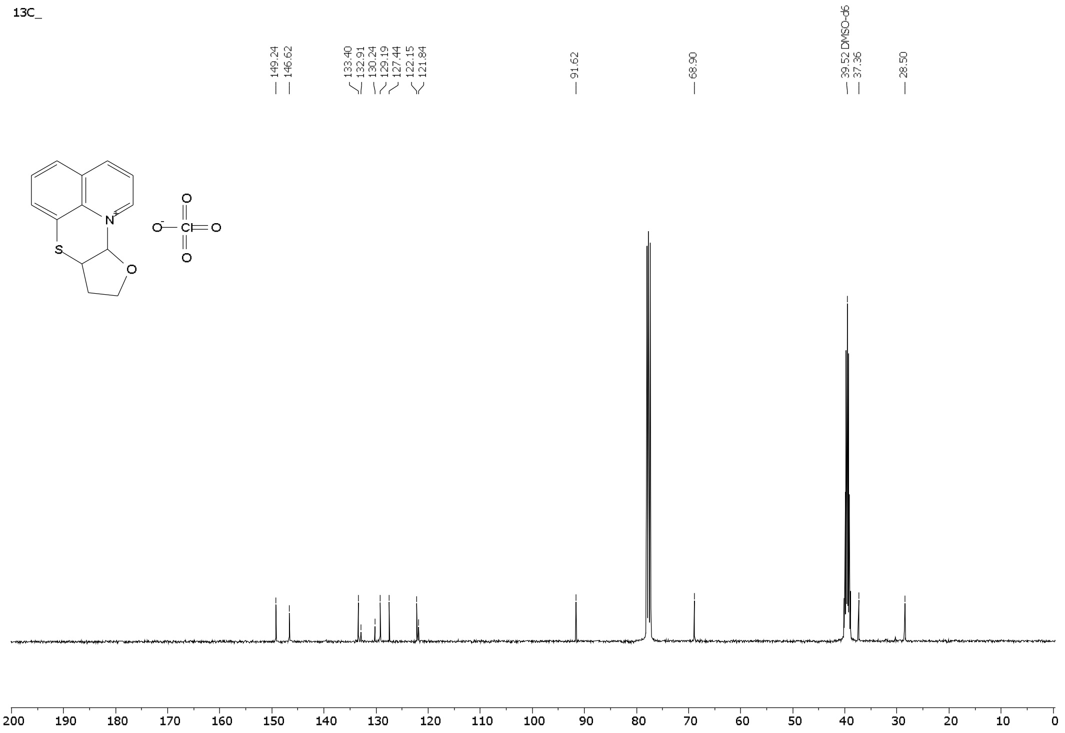

^{13}C-NMR (DMSO-*d_6*/$CDCl_3$, 1:1) spectrum of 7a*H*,8*H*,9*H*,10a*H*-furo[2',3':5,6][1,4]thiazino[2,3,4-*ij*]quinolin-11-ium perchlorate (13)
